# Advances in breast cancer diagnosis: a comprehensive review of imaging, biosensors, and emerging wearable technologies

**DOI:** 10.3389/fonc.2025.1587517

**Published:** 2025-06-18

**Authors:** Abdul Qadeer Khan, Manahil Touseeq, Sara Rehman, Maria Tahir, Malaika Ashfaq, Emaan Jaffar, Saadullah Farooq Abbasi

**Affiliations:** ^1^ Department of Biomedical Engineering, Riphah International University, Islamabad, Pakistan; ^2^ Department of Electronic, Electrical and Systems Engineering, University of Birmingham, Birmingham, United Kingdom

**Keywords:** breast cancer, imaging, biosensors, wearable technologies, artificial intelligence

## Abstract

Breast cancer has been the most frequent diagnosed cancer and the leading cause of cancer-related deaths among women worldwide, mainly due to delayed detection. Early diagnosis significantly improves prognosis and long-term survival rates. Various techniques, including imaging, sensors, and molecular biotechnology, have been developed to facilitate early detection. This review provides a comprehensive analysis of these diagnostic techniques, emphasizing precision, patient comfort, and cost-effectiveness. Additionally, it explores the emerging role of wearable technologies, such as smart bras and real-time monitoring devices, in revolutionizing breast cancer detection. The review concludes by discussing the limitations of current diagnostic methods and proposing future directions for enhancing early detection and improving patient outcomes.

## 1 Introduction

Breast cancer is the most diagnosed malignancy and one of the leading causes of cancer-related deaths among women worldwide (1). Breast cancer occurs when normal breast cells undergo genetic mutations, leading to uncontrolled growth, known as neoplasia. If not detected early, cancerous cells can invade surrounding tissues and spread to distant organs, complicating treatment and increasing the risk of fatality. To improve the prognosis and survival rates of breast cancer, early detection is essential. Early detection of breast cancer greatly improves long-term results by increasing the likelihood of successful treatment and breast-conserving surgery. Due to localized disease management and less aggressive treatment requirements, studies have demonstrated that patients diagnosed at earlier stages have higher survival rates than those diagnosed at advanced stages (1, 2). By reducing the need for extensive therapies like chemotherapy and radiation, early detection also lessens the financial and emotional burden on patients (2).This emphasizes how crucial it is to keep working to improve breast cancer screening tools and encourage routine examinations for early detection. Breast cancer is a significant global health concern, with rising incidence and mortality rates. From 2008 to 2017, new cases increased by 6%, reaching 11.7% in 2020, when approximately 685,000 women died, and 2.3 million new cases were reported. Early detection greatly enhances survival rates, with nearly 90% survival for early-stage diagnoses (2, 3). Given global population growth, experts estimate that by 2050, the number of new breast cancer cases will rise to approximately 3.2 million annually (4). Notably, breast cancer is increasingly affecting younger populations, raising concerns about its detection and management. Several risk factors, such as age, family history, lifestyle, unregulated use of medications like oral contraceptive pills (OCPs), and more, contribute to this trend. Recent studies indicate that prolonged use of hormone replacement therapy (HRT) for over 5–7 years increases breast cancer risk (3, 4). Similarly, Wang et al. presented that elderly woman, with higher BMI, have increased cancer risk compared to those with lower BMI (5). Additionally, alcohol can also increase the risk of estrogen-positive breast cancers (6).

Breast cancer poses a significant challenge due to the lack of early symptoms, often resulting in late-stage diagnoses. Factors like limited awareness, inadequate healthcare access, and infrequent screenings contribute to this issue. Early and accurate diagnosis is vital for survival. While traditional screening methods, such as mammography and clinical breast examinations, remain standard, they have limitations, including high false-positive rates and lower sensitivity for women with dense breast tissue. Breast cancer rates are notably higher in premenopausal women. While it is rare in those under 40, it has recently raised concerns. Due to the density of their breast tissue, premenopausal women are usually not included in screening programs or recommended to have mammograms (5–8).

**Figure 1:**
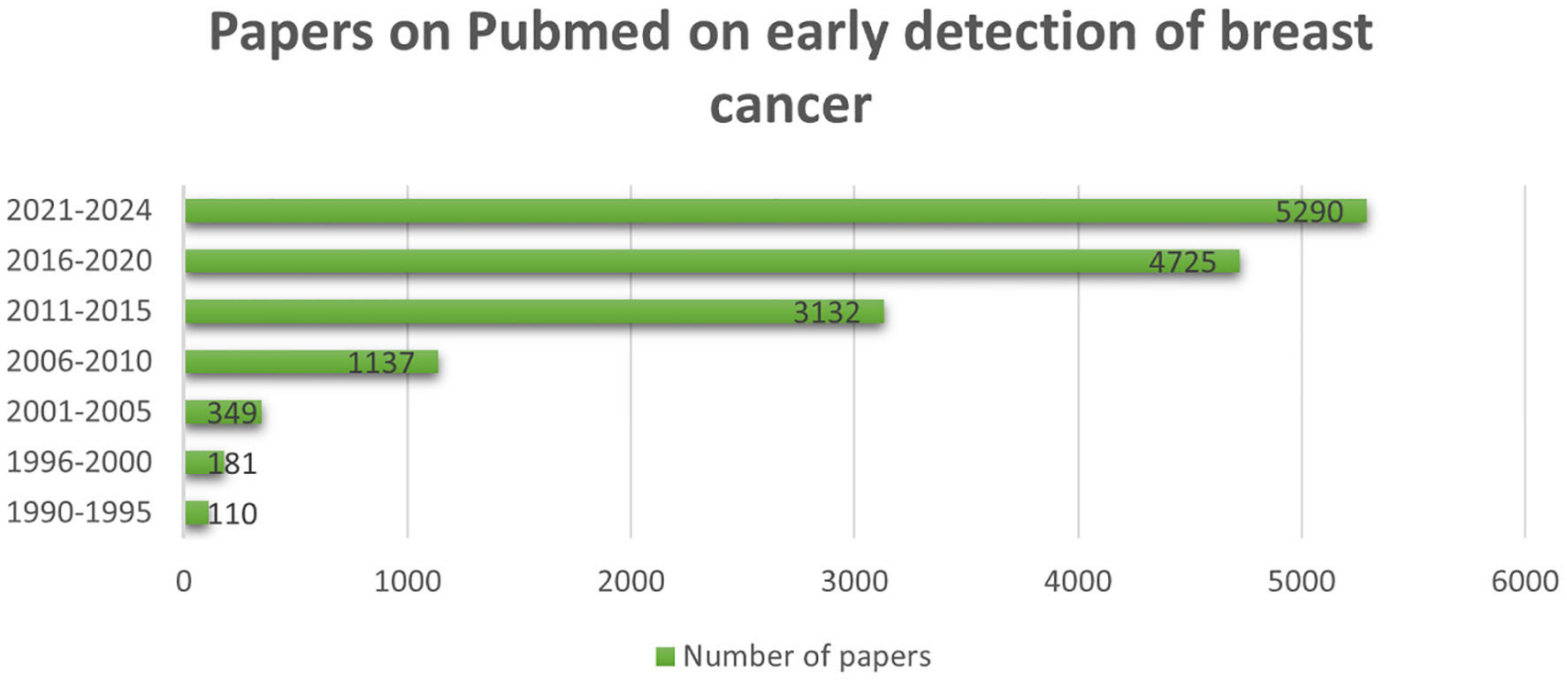
The annual trend of research publications related to breast cancer, as evidenced in PubMed, demonstrates a significant increase in scholarly interest over the years. (1990-2024).

**Figure 1** presents a year-by-year analysis of research publications on breast cancer available in PubMed. Breast cancer in men is rare, accounting for less than 1% of all breast cancers worldwide and approximately 1% of all malignancies in men (9, 10). The most affected region in male breast cancer (MBC) is the nipple/areola area (11–15). Due to its rarity, early diagnosis remains a challenge, and there are limited therapeutic strategies and awareness programs specifically targeted at MBC. Consequently, treatment options for male breast cancer remain suboptimal (16). Male breast cancer (MBC) has distinct biological differences compared to female breast cancer. MBC is almost always hormone receptor-positive (HR+) and often associated with BRCA2 germline mutations, which increase the risk of aggressive breast cancer in men (17). Additionally, germline pathogenic variants (PVs) in the BRCA1/2 genes have been linked to an elevated risk of BC in both men and women. Multigene panel testing is increasingly used to assess breast cancer risk, allowing for the detection of pathogenic variants beyond BRCA1/2 (18).

**Figure 2:**
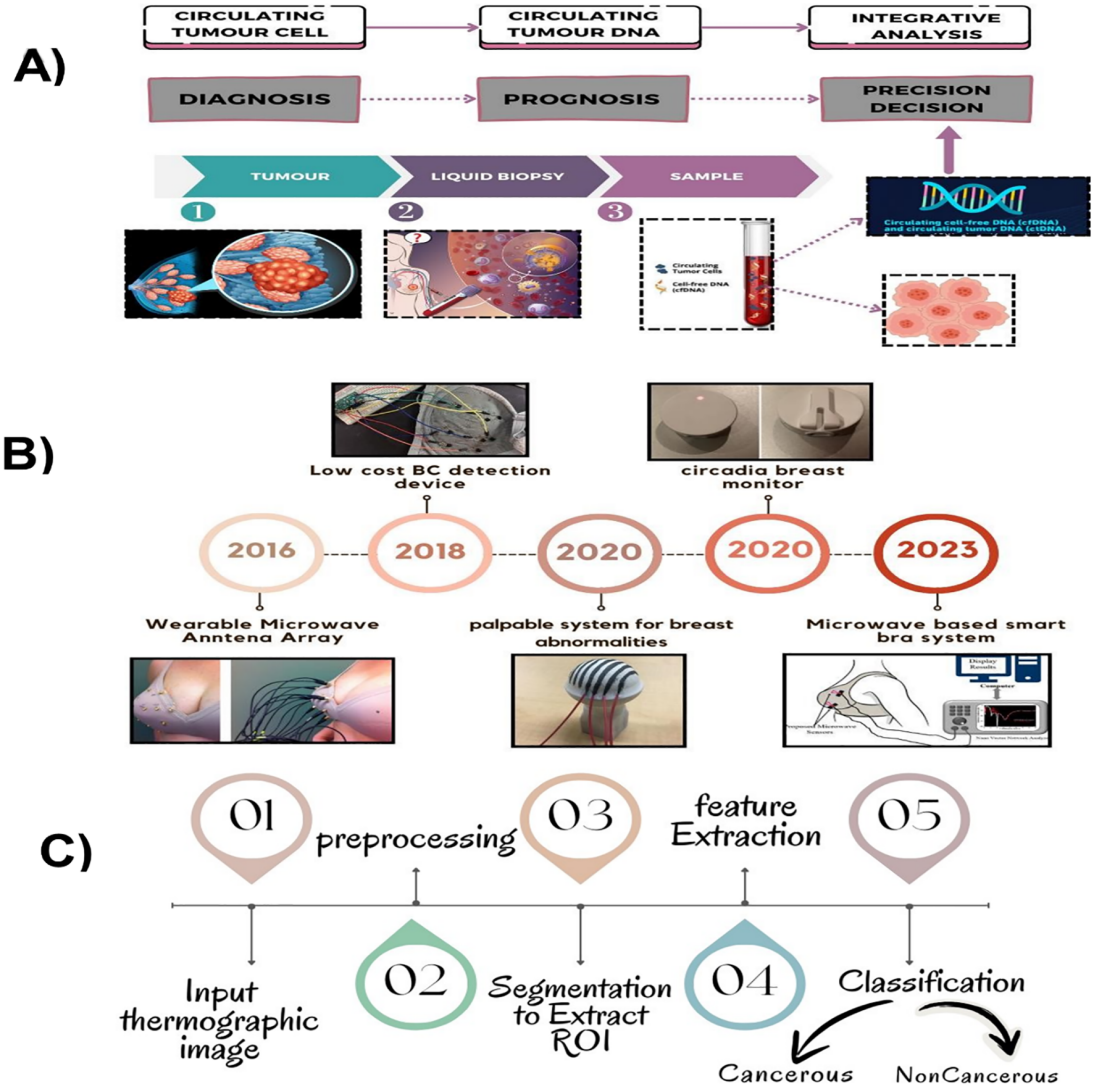
A summary of the diagnostic process for breast cancer that includes: **(A)** a liquid biopsy method that shows the extraction of biomarkers; **(B)** a timeline of sensor developments used in detecting technologies; and **(C)** a flow diagram that shows the AI-based breast cancer detection framework.

**Figure 2** shows the advances in breast cancer diagnosis: **Figure 2A** represents the basic process of the liquid biopsy procedure; 2B shows the sensors and embedded devices developed for the early detection of breast cancer, year by year and finally, 2C shows the flow process of breast cancer detection using AI. Mammography and clinical breast examination are the two most frequently used methods for breast screening (7, 8). To obtain a tissue sample for further histopathological diagnosis, a needle biopsy is essential. There are three methods of needle biopsy: vacuum-assisted breast biopsy (VABB), core needle biopsy (CNB), and fine-needle aspiration cytology (FNAC) (19). Although mammography is considered as the gold standard for diagnosing breast cancer, it has several limitations, including high false positive rates, limited effectiveness in cases of dense breasts, and the use of ionizing radiation (20). To address this issue, the integration of artificial intelligence (AI) with mammography is crucial for screening purposes. With thorough study and testing, these AI systems could potentially take over the role of radiologists in reading mammograms. However, adequate preparation and high-quality data are necessary for AI systems to function effectively. AI can be incorporated into regular screening procedures with the right investigation and validation (21). The proposed study explores screening methods that can help detect this deadly disease with minimal harm. Research on screening techniques should aim to reduce the number of undetected advanced cancers, as well as unnecessary biopsies and follow-up procedures. When discussing ultrasound, it has a sensitivity of 80%, but it is not suitable for imaging bony structures (9, 22). Thermography can be used to detect breast cancer in its early stages, potentially reducing the need for unnecessary biopsies in breast cancer screening (10, 11). However, a drawback of thermography is its inability to identify the specific cause of an increase in breast temperature. This is because mastitis, an inflammation of the breast tissue, can also lead to an increase in breast temperature. The risk of developing breast cancer increases by 2% for each x-ray exposure (12). Many publications are available for diagnosing BC, but few specify which approach is best for a certain subset of BC patients (13).

This review provides an in-depth analysis of the current and emerging breast cancer diagnostic techniques, with a focus on their advantages, limitations, and potential for improving early detection. We discuss various imaging modalities, including reflective optical imaging, microwave imaging, and ultrasound, as well as sensor-based detection techniques such as thermography, piezoresistive, near-infrared, and bioimpedance spectroscopy-based sensors. Additionally, we explore biosensor technologies, including optical biosensors (colorimetric, fluorescence, surface plasmon resonance imaging [SPRi], surface-enhanced Raman spectroscopy [SERS], and electrochemiluminescence [ECL] biosensors), electrochemical biosensors (field-effect transistor [FET], electrochemical impedance spectroscopy [EIS], and voltametric techniques), and other biosensors such as quartz crystal microbalance (QCM) and photoelectrochemical (PEC) biosensors. The review concludes by discussing the limitations of existing diagnostic techniques and potential future directions for improving breast cancer detection, with an emphasis on precision, accessibility, and patient-centric care.

## 2 Materials and methods

### 2.1 Goal of the review

The primary goal of this study is to enhance the understanding of breast cancer diagnosis by evaluating various early detection techniques, including biomarkers, biosensors, artificial intelligence (AI), sensors, and imaging technologies. This review explores the role of tumor markers, their detection methods, the advancements in biosensor technology, and the application of AI in improving diagnostic accuracy.

Our analysis provides a comprehensive assessment of past and emerging diagnostic approaches, emphasizing recent developments and breakthroughs in biosensors, AI, imaging, and sensor-based technologies. We present a balanced evaluation of each technique, discussing its advantages and limitations. Special attention is given to sensor-based methods, which offer affordable, accessible, and non-invasive breast cancer screening solutions. Additionally, figures and graphical illustrations are incorporated throughout the review to visually represent key findings and the relationships between different diagnostic methodologies.

### 2.2 Data sources

A systematic search strategy was employed to identify relevant research articles published between 2000 and 2024, with a primary focus on studies from 2015 to 2024. The databases searched included: PubMed; Springer; IEEE Xplore; ScienceDirect; Gray Literature, including Google Scholar. To ensure a comprehensive literature review, keyword searches were performed using the following search terms: “Early breast cancer detection”, “Early breast cancer detection through biomarkers”. “Early detection of breast cancer using sensors”, “Breast cancer screening techniques”, “Artificial intelligence techniques for early breast cancer detection”. The wildcard symbol (*) was used to retrieve variations of keywords, and Boolean operators (“AND” “OR,” “NOT”) were applied to refine the search.

### 2.3 Inclusion and exclusion criteria

Only peer-reviewed articles published in English were included.

Articles with similar findings and methodologies were excluded to avoid redundancy.

Non-English publications and studies without full-text access were omitted.

From an initial pool of 30,991 research articles retrieved from PubMed, filtering for free full-text availability and relevance to biosensor-based detection resulted in 98 selected papers. Similarly, filtering for sensor-based detection identified 94 studies, and the same method was applied for AI and imaging-based detection approaches.

**Figure 3:**
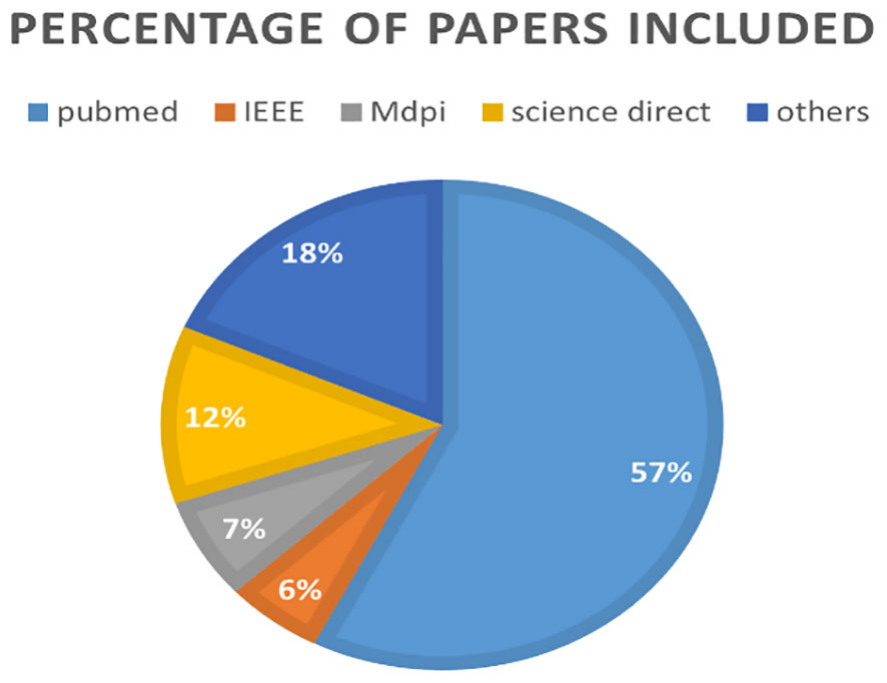
The distribution of the shortlisted papers on early breast cancer detection from PubMed, IEEE, MDPI, ScienceDirect, and other databases.

Applying the same search strategy on Google Scholar, we identified a total of 238 eligible papers, all of which were available in PDF or free-text format and aligned with the scope of this review. The distribution of the shortlisted papers on early breast cancer detection from PubMed, IEEE, MDPI, ScienceDirect, and other databases in **Figure 3**.

#### 2.3.1 Biomarkers

##### 2.3.1.1 Tumor marker

A group of active compounds, called tumor markers, are formed when the body tissue or tumor interacts with them. These molecules can indicate the presence and progression of a tumor. Various factors like tumor size, mass, expression level, breakdown, excretion rates, blood supply, and resistance to medication can affect the concentration of tumor markers at different stages. Examples of commonly used indicators for breast tumors include human epidermal growth factor receptor 2 (HER2), progesterone, and estrogen receptor. Additionally, ongoing research is exploring the role and potential of newly developed biomarkers in the detection and management of breast cancer (14, 15). **Table 1** explains the Overview of tumor markers used in breast cancer detection.

**Table 1:** Overview of tumor markers used in breast cancer detection.

Tumor marker	How it works	Pictorial representation	Reference
Estrogen Receptors	Estrogen receptor (ER) binding in cells is essential for diagnosing metastatic breast cancer, prognosing, and assessing endocrine therapy suitability. A positive ER result indicates effective treatment, while a negative result does not.	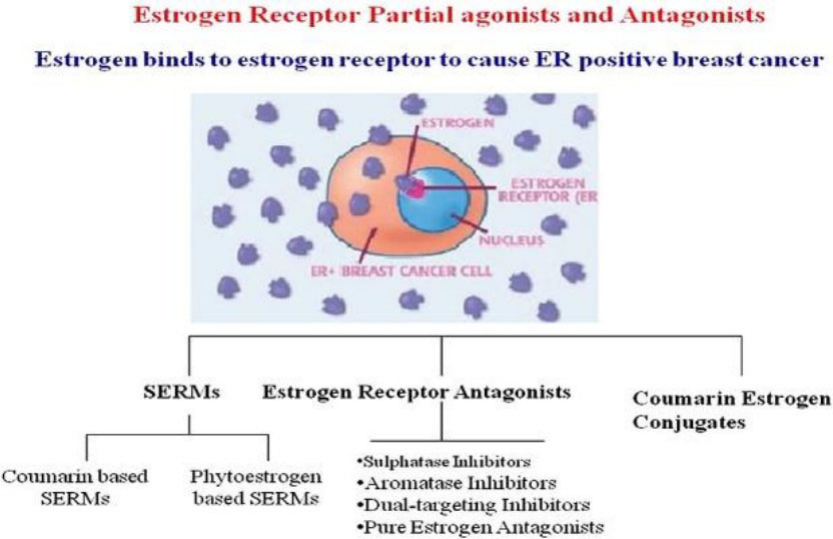	(38)
Progesterone Receptors	PR, a hormone receptor, signals ER activity, affecting cellular gene regulation. PR-positive breast cancer patients account for 65-70%, necessitating re-detection to assess prognosis.	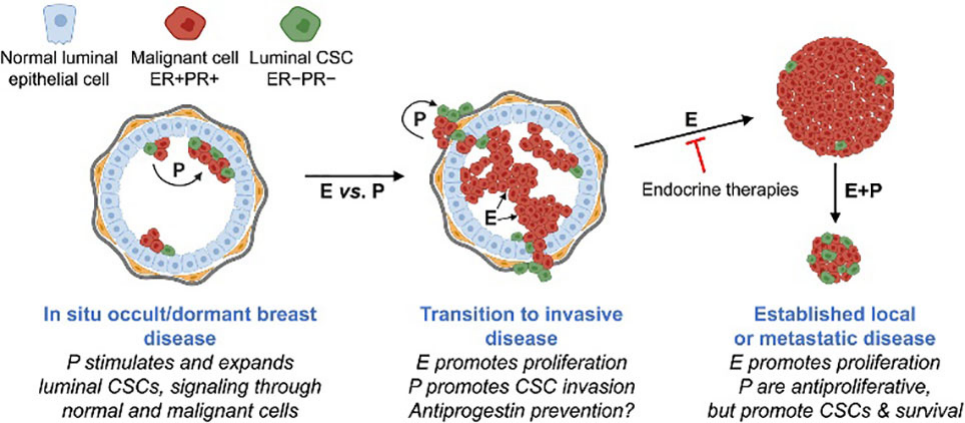	(39)
The Biomarker of Triple Negative Cancer (TNBC)	NBC, a subtype of breast cancer, has poor prognosis and lower survival rates, with biomarker surveys identifying VEGF as a marker for targeted therapy.	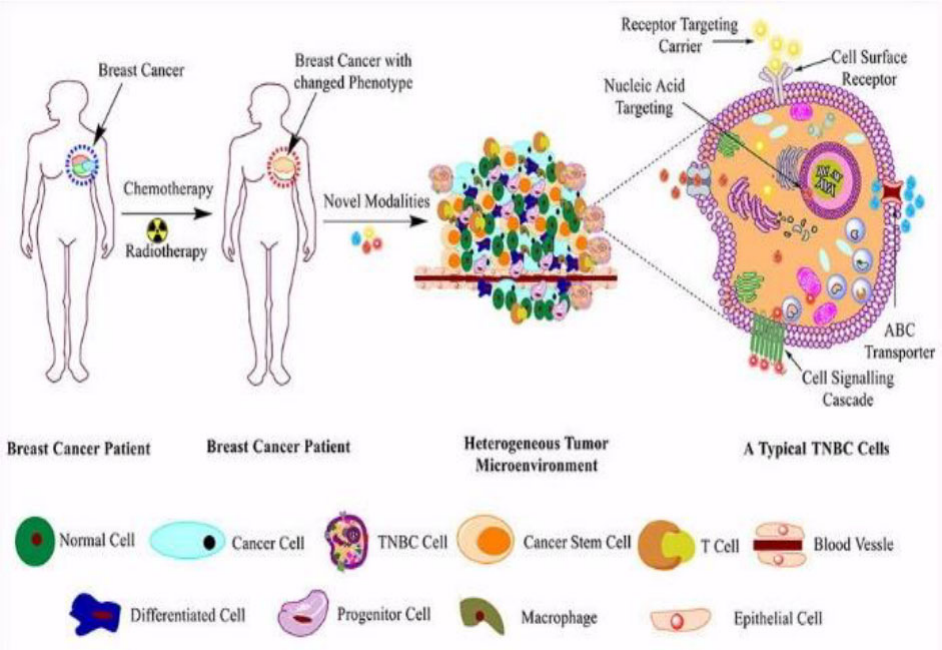	(40)
Human epidermal growth factor Receptor 2	HER2 gene promotes tumor growth, affecting prognosis. HER2-targeted therapy is used in HER2-positive patients, with HER2 levels negatively correlated with ER and PR levels.	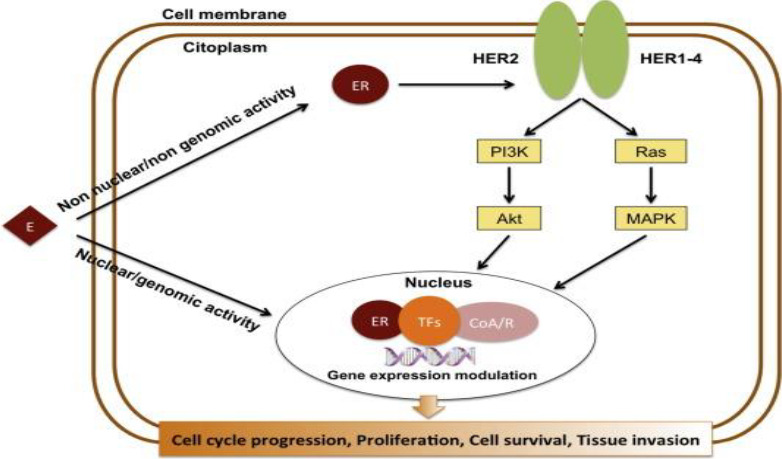	(41)
Emerging Tumor Marker	Researchers are exploring new tumor indicators, including proteins, nucleic acids, cancer cells, and other types of cells, in addition to the three common clinical breast cancer markers.	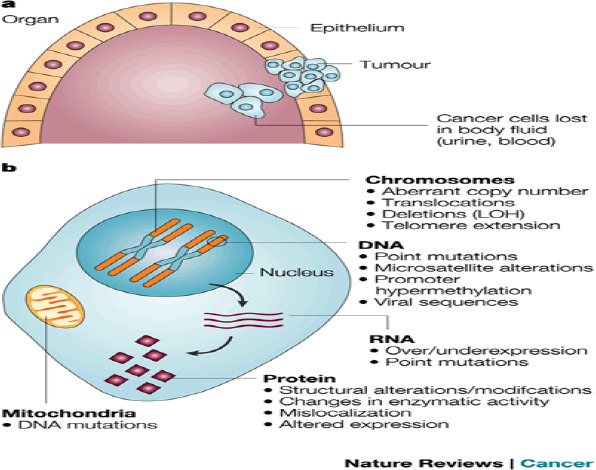	(42)

When determining if an organism or pathogenic process is normal or if therapeutic intervention is necessary, a biomarker provides an objective measurement (16). Stated differently, biomarkers are chemical indications of disease status that help distinguish between a normal tumor and a cancerous one (17, 18). Therefore, biomarkers provide insights into the onset and progression of cancer in the body. Body fluids such as blood, urine, and saliva can be used as analytes in sensor development because they contain biomarkers (23–25).

###### 2.3.1.1.1 Estrogen receptor

A protein molecule called the estrogen receptor (ER) binds to estrogen in cells only (26). Cytoplasm, nucleus, or cell membrane can all have estrogen receptors. The conventional nuclear receptor is found in the nucleus, and following translation, its protein is momentarily translated into the cytoplasm, where it is detectable (27). As estrogen diffuses into the nucleus, it attaches to its nuclear receptor, activating a mechanism that controls gene regulation and the transcription of genes downstream. Estrogen receptor detection serves as a diagnostic tool for metastatic breast cancer, helps in prognostication, and assesses if a patient is appropriate for endocrine therapy. It does this by binding to the estrogen receptor in the patient. Consequently, if a patient has been shown to have estrogen receptors, this indicates that the patient may be a good candidate for endocrine therapy. For individuals who test positive for ER, endocrine treatment is an effective way to stop tumor progression (28). Patients who test negative for ER cannot benefit from the same treatment. Unmistakable data suggests that endocrine therapy is not beneficial for patients whose tumors do not express ER (29).

Upon estrogen binding (E2) or phosphorylation (P) by cellular kinases following growth factor (GF) receptor stimulation, ERα is activated and translocates into the nucleus. Once there, ERα can bind DNA directly or indirectly through estrogen-responsive elements (EREs) or by binding to other transcription factors such as AP1 or SP1, which bind DNA through serum-responsive elements (SREs). This genomic action of ERα regulates the transcription of target genes. Additionally, ERα can be anchored to the membrane and interact with G proteins (Ga) or GF receptors, leading to non-genomic activity such as the production of second messengers (cyclic adenosine monophosphate, cAMP) and stimulation of signaling pathways involving PI3K/AKT or Ras/MAPK. This non-genomic activity eventually leads to the activation of transcription factors (TFs) involved in the regulation of cell proliferation and survival (30).

###### 2.3.1.1.2 Progesterone receptor

The progesterone receptor (PR) is a hormone receptor, like the ER. ER activates PR, and PR activation is a signal of ER activity (31). The interaction between PR and chromatin changes the binding position of ER and chromatin and then leads to a change in cellular gene regulation from proliferation to cell cycle arrest, apoptosis, and differentiation (27). PR-positive patients account for approximately 65–70% of breast cancer patients, and PR-positive patients are rarely concurrently ER-negative (31). Therefore, in strongly PR-positive and ER-negative patients, re-detection of ER is necessary to exclude the possibility of a false-negative result (28, 32). The main purpose of PR detection is to assess the prognosis of ER-positive patients (31).

###### 2.3.1.1.3 Human epidermal growth factor receptor 2

The human epidermal growth factor receptor 2 (HER2) gene is one of the most studied breast cancer proto-oncogenes (15). HER2 promotes tumor growth by activating MAPK and PI3K/AKT signaling pathways, which in turn increase cell proliferation, invasion, and metastasis (27) In the absence of systemic therapy, HER2 gene amplification or protein expression is associated with a poor prognosis.HER2 levels were found to be negatively correlated with ER and PR levels (15). HER2-positive patients account for approximately 15–20% of breast cancer patients. In clinical practice, HER2-targeted therapy is used in HER2-positive patients and HER2 is used as a prognostic indicator. As with ER therapy, HER2- targeted therapy works only in HER2-positive patients but not in HER2-negative patients (27).

###### 2.3.1.1.4 The biomarker of triple negative cancer

TNBC, a subtype of breast cancer that lacks ER, PR, and HER2 expression, accounts for 15-20% of patients. Triple-negative breast cancer (TNBC) has a worse prognosis and a lower survival rate. Currently, the most important treatment is cytotoxic chemotherapy. Further classification of TNBC is needed for more targeted therapy. A survey of biomarkers associated with TNBC has identified several biomarkers that can stratify patients for molecular therapy. VEGF, a key signaling factor, is highly expressed in 30-60% of TNBC patients and targeted anti-VEGF therapy improves treatment outcomes (33, 34). Binding Androgen in cell depends upon a hormone called as Androgen receptor(AR), this binds the transcription factor as well as control gene Expression. AR simulate proliferation as well dedifferentiation and induce cell death and apoptosis. The expression of AR is related to the biological behaviors of triple-negative breast cancer and plays a role in endocrine therapy and prognostic prediction (35, 36).

###### 2.3.1.1.5 Emerging tumor marker

Researchers are now focusing on newly discovered tumor indicators in addition to the three typical clinical breast cancer tumor markers discussed earlier. These new indicators can be classified as proteins, nucleic acids, cancer cells, and other types of cells (37). One type of active material that can show the presence and progression of a tumor is a tumor marker. Finding tumor markers can be a useful tool in the diagnosis and management of breast cancer. Some of the drawbacks of the traditional tumor marker detection approaches are high equipment costs, labor-intensive procedures, and low sensitivity.

#### 2.3.2 Biosensors

**Figure 4:**
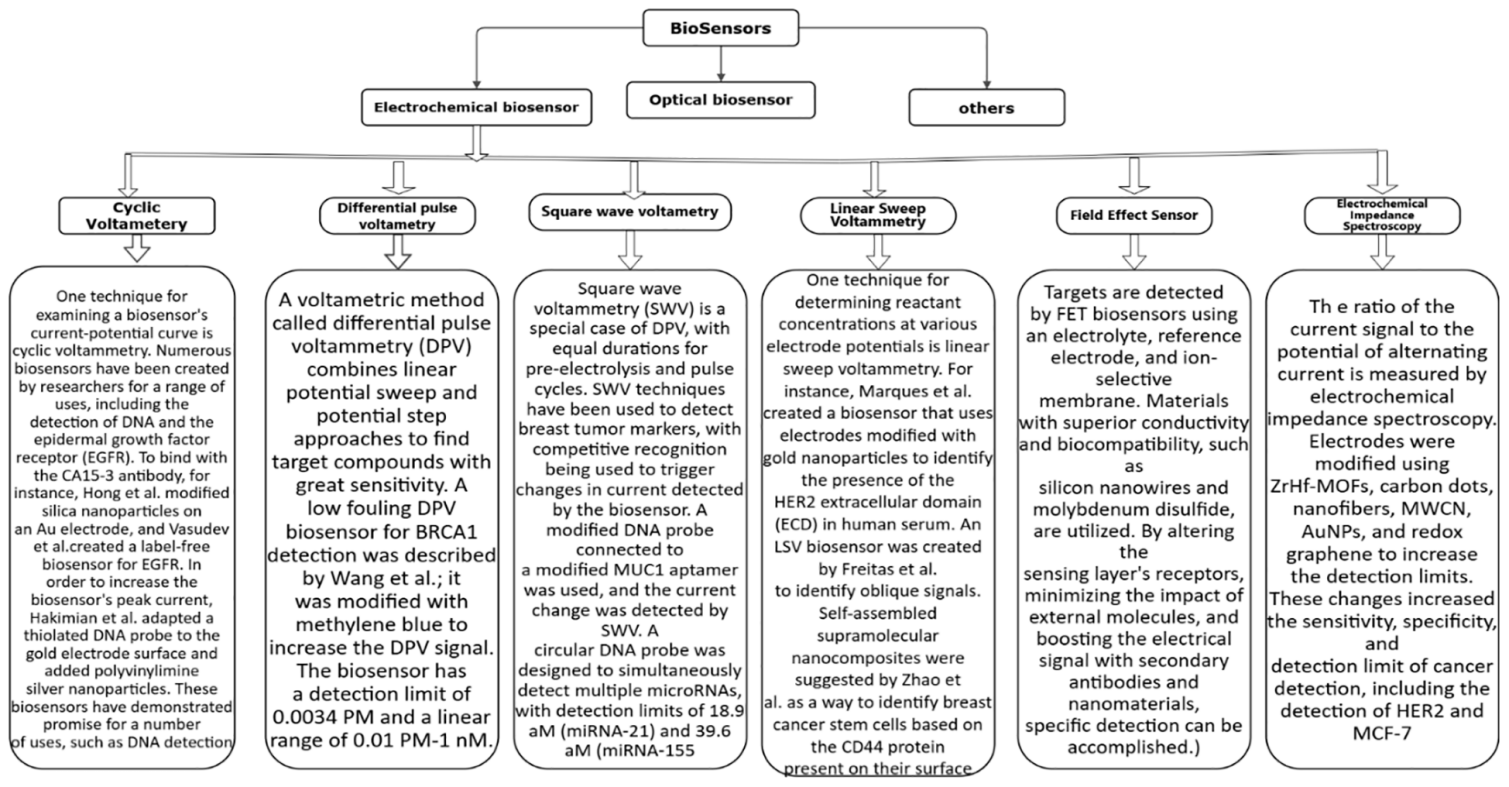
A brief overview of the types of electrochemical biosensor utilized in breast cancer detection.

**Figure 5:**
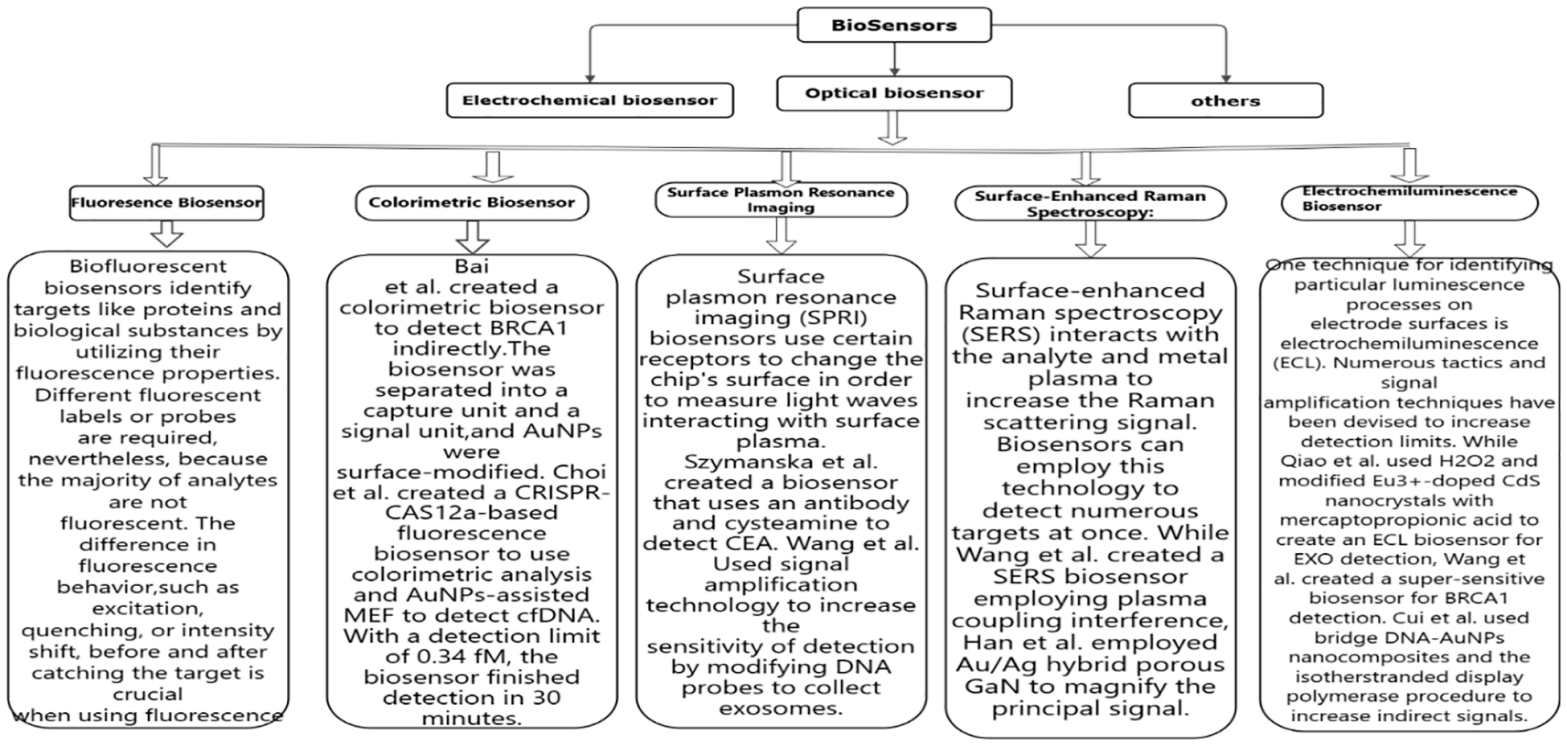
A brief overview of the types of optical biosensors used in breast cancer detection.

**Figure 6:**
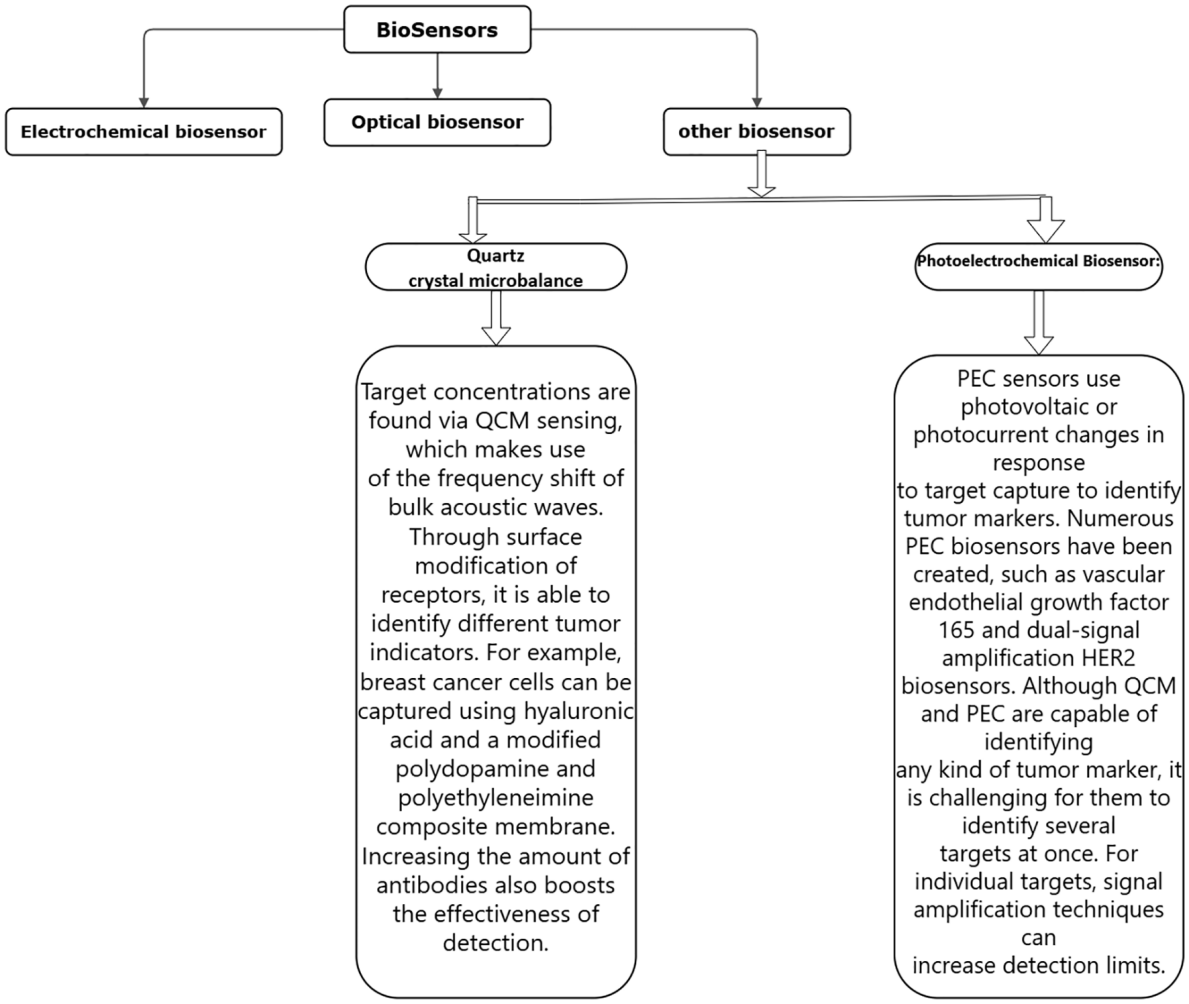
A brief overview of the types of other biosensors used in breast cancer detection.

Depending on the detecting signal and detection technique, biosensors can be categorized as electrochemical, optical, or other types (43–47). Numerous biosensors for identifying breast tumor indicators have been created in recent years by researchers. This study reviews the advances made in the development of electrochemical biosensors, optical biosensors, and other forms of biosensors for breast tumor indicators. **Figures 4**–**6** provides a brief overview of the types of electrochemical, optical and other biosensors utilized in breast cancer detection.

##### 2.3.2.1 Electrochemical biosensor

Electrochemical biosensors rely on the detection of electrochemical processes occurring on electrode surfaces to determine target concentration. Thorough explanation of the types of electrochemical biosensors used to detect breast cancer (**Table 2**).

**Table 2:** Thorough explanation of the types of electrochemical biosensor used to detect breast cancer.

Name	Type	Target	Detection Limit	diagrams	Reference
Electrochemical Biosensor	CV	CA153 EGFR miRNA-155	0.64 U mL−1–1 pg mL−1 2×10^−2M	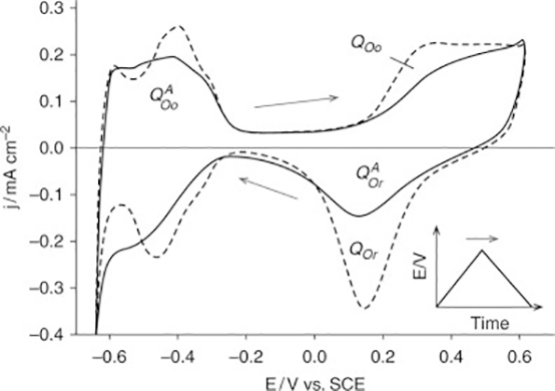	(48) (49) (50)
	DPV	BRCA1 CA15-3 BRCA1 let-7a miRNA-21	0.0034 pM 3.34mUml^-1 3.01 × 10−16 M (let-7a) 8.2fM (miRNA-21)	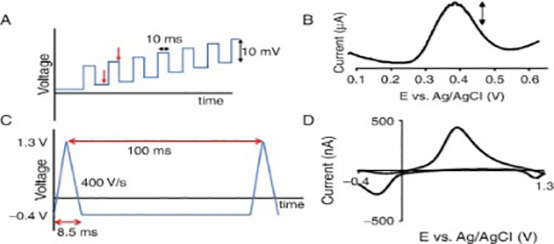	(51) (52) (53) (54)
	SWV	MUC1 miRNA-21 miRNA-155	0.33 pM 39.6 aM 18.9 aM	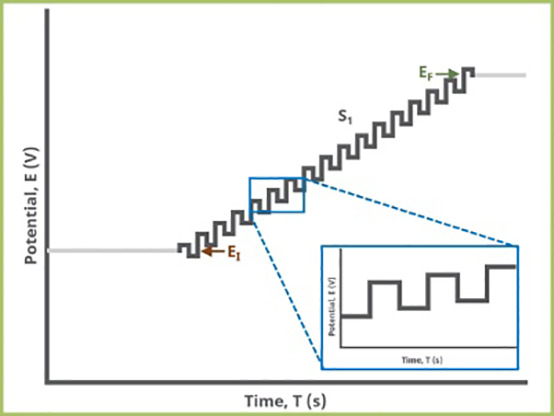	(55) (56)
	LSV	HER2 HER2-ECD CD44 CD44 positive cells	0.16 ng mL 1 4.4 ng mL 1 2.17 pg. mL−1–8 cells mL−1	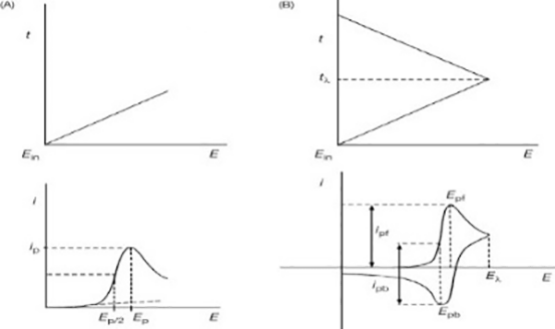	(57) (58) (59) (60)
	EIS	HER 2 MCF-7 cell MUCI BRAC1	23 cells mL−1 2.7 nM	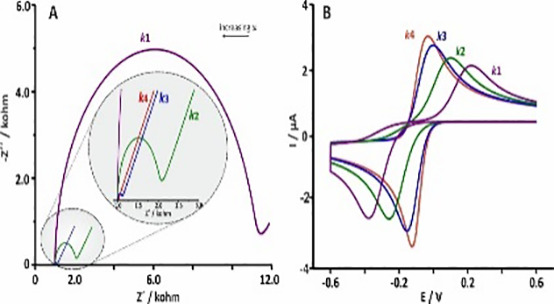	(61) (62) (63)
	FET	miRNA-155 CEA	0.03 fM	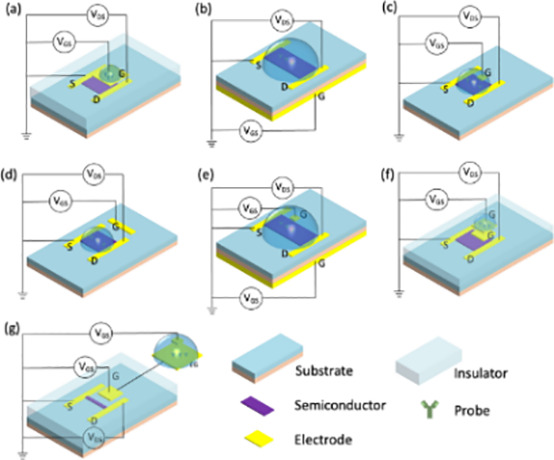	(64) (65) (66) (67)

##### 2.3.2.2 Optical biosensor

Refractive index, resonance, wavelength, intensity, and other optical changes on sensing layers are used by optical biosensors to identify targets.

Various optical sensors, such as colorimetric, fluorescence, SPRi, SERS, and ECL biosensors, have shown varying linear ranges and detection limits for distinct biomarkers. With a linear range of 0.03–6 ng mL−1, fluorescence biosensors have identified CEA at 7.9 pg mL−1 in water and 10.7 pg mL−1 in human serum samples. Using fluorescence, the detection limit of miRNA-21 is 0.03 fM, with a linear range of 0.1–125 fM (68, 69), The linear ranges of colorimetric biosensors are 10−12–10−18 M and 1 fM–100 pM, respectively, and they have detected BRCA1 at 10−18 M and 0.34 fM (70, 71), SPRi biosensors have identified HER2-positive EXO at 8280 exosomes µL−1, with a range of 8280–33,100 exosomes µL−1, and CEA at 0.12 ng mL−1, with a linear range of 0.40–20 ng mL−1. miR-K12-5-5p was discovered by SERS biosensors at 884 (72–76).

Because of their remarkable sensitivity to biomolecular interactions, PCF SPR (Photonic Crystal Fiber Surface Plasmon Resonance) biosensors have become promising instruments for the early detection of breast cancer. Ultra-low concentration detection is made possible by these biosensors, which pick up on minute changes in refractive index when breast cancer biomarkers adhere to metal surfaces. According to recent research, resonance shifts, and detection efficiency can be enhanced by optimized PCF designs that incorporate modifications to geometry and metal layering (77). In order to address the issue of environmental sensitivity, additional developments in nanomaterials and optical configurations improve signal stability and accuracy. PCF SPR biosensors are anticipated to transform real-time, non-invasive breast cancer detection as research advances (78).

##### 2.3.2.3 Other types of biosensors

Finally, it should be noted that QCM and PEC can identify any kind of tumor marker. The primary purpose of the QCM biosensor’s signal amplification is to increase the mass change of the chip surface. The PEC biosensor uses signal amplification to increase the photovoltaic and photocurrent changes brought on by the target. While signal amplification techniques can be used to increase the detection limits of QCM and PEC sensors for a single target, these sensors struggle to detect many targets at once (79–83).

###### 2.3.2.3.1 Innovative approaches for biosensor

The detection approach and the detecting device are the biggest obstacles for biosensors. Detection techniques typically find it challenging to handle biomolecules in challenging situations. For instance, the environment frequently affects the activity and shelf life of biomolecules. The majority of biosensors are not sufficiently integrated and compact, making it impossible for them to detect several targets at once at the device level. To overcome these obstacles, the coupling of microfluidic chips with biosensing and molecularly imprinted polymers (MIPs) holds considerable promise.

###### 2.3.2.3.2 Summary of tumor marker and biosensors

Tumor indicators play an important role in breast cancer diagnosis and treatment, however no marker can effectively predict breast cancer before clinical symptoms appear. HER2-targeted biosensors allow for real-time detection of overexpressed HER2 proteins, often linked to aggressive types of breast cancer. This early detection enables quick initiation of HER2-specific therapies, improving treatment response and greatly increasing patient survival rates outcomes (84). Biosensor development has obstacles in detecting several targets at the same time, as most biosensors can only detect one target. Biosensors, such as antibodies, DNA probes, and aptamers, can give excellent sensitivity and specificity in laboratory settings. However, sensitivity in human serum samples declines due to chemical variables, which is especially critical for whole blood samples. To improve sensitivity and specificity, biosensors must improve their detecting technique and technology. The detection method seeks to improve both sensitivity and specificity, while the technology enables simultaneous detection of many objects. The usefulness of biosensors in practical applications for breast cancer detection has been shown by recent clinical validation studies. By tracking changes in refractive index as a result of antigen-antibody interactions, electrochemical and optical biosensors, such as PCF-SPR (Photonic Crystal Fiber Surface Plasmon Resonance) biosensors, have demonstrated high sensitivity in identifying breast cancer biomarkers. Repeated biopsies may not be necessary thanks to these devices’ potential for quick and non-invasive diagnosis (77). The stability and accuracy of biosensors have been further improved by developments in material science and nanotechnology, which have increased their clinical relevance. Recent advancements in biosensor technology have improved the detection of breast cancer due to their high sensitivity and real-time biomarker analysis. Although they don’t have constant monitoring, non-wearable biosensors offer remarkable accuracy in regulated settings. On the other hand, implantable biosensors provide real-time, *in vivo* tracking, which increases diagnostic accuracy. However, they pose challenges in terms of invasiveness and patient comfort. The requirement to balance accuracy and usability must continue to drive biosensor research in the future. **Table 3** presents a detailed comparison of non-wearable and implantable biosensors for breast cancer detection, evaluating various clinical and operational parameters. Below, the table outlines a comparative study of the accuracy and patient outcomes concerning comfort for both biosensor types (77, 94).

**Table 3:** A comparative analysis of non-wearable and implantable biosensors for breast cancer detection (85).

Feature	Non-Wearable Biosensors	Implantable Biosensors	References
Placement	External (used in labs or diagnostic facilities)	Under the skin or internally placed through minimally invasive procedures	(86)
Monitoring Frequency	Periodic testing (e.g., blood samples, imaging visits)	Continuous real-time tracking of physiological or biochemical markers	(87)
Sample Type	Blood, serum, saliva, or tissue biopsies	Interstitial fluids, internal biomarker levels (e.g., HER2, pH, oxygen)	(88)
Detection Method	Electrochemical, optical (PCF_SPR), or immunoassays	Embedded microelectrodes, nanomaterial-based sensing elements, wireless signal transmitters	(89)
Invasiveness	Non-invasive or minimally invasive	Minimally invasive (requires implantation under skin or tissue)	(90)
Diagnostic Accuracy	High for certain biomarkers (e.g., HER2, CA 15-3); moderate for others	High specificity in pilot studies; early-stage detection potential	(86, 91)
Patient Comfort	Generally comfortable; requires clinic visits	Moderate; possible discomfort due to implantation or foreign body sensation	(92)
Power Supply	Externally powered (lab equipment)	Battery-powered or wirelessly powered via telemetry	(86)
Usage Context	Hospitals, diagnostic centers	Chronic condition monitoring, clinical research environments	(91)
Development Stage	Mature and widely adopted	Experimental, under preclinical or clinical testing	(93)

New nanomaterials, second antibodies, and indirect signal detection methods can help detect biomolecules. Sample pretreatment and MIPs can help with background interference and biomolecules’ trouble in hostile conditions. Combining microfluidic chips with biosensing can boost overall performance and multi-target detection. These new technologies can be marketed on a wide scale, altering the current detection paradigm and potentially leading to a shift. Biosensor technology must continue to advance in order to reach its full potential in the detection of breast cancer. This development calls for attention to be paid to both signal processing algorithms and biosensor materials. In terms of materials, research is still needed to create new materials with higher sensitivity so that biomarkers can be found at lower concentrations and an earlier diagnosis can be made. In order to reduce false positives, materials that are made to specifically target particular biomarkers and minimize interference from other substances are necessary for improved specificity. To guarantee accurate and consistent readings, biosensors must also show improved stability over time and in a variety of scenarios. Biocompatibility is another important factor for implantable sensors. It is crucial to develop complex signal processing algorithms concurrently with material advancements. These algorithms ought to be able to extract pertinent features from complex sensor data in order to identify subtle changes suggestive of cancer, effectively reduce noise in sensor signals, and adjust for sensor drift to preserve long-term reliability. The potential for further improving signal processing capabilities and raising the overall accuracy of breast cancer detection is high when artificial intelligence and machine learning techniques are combined.

#### 2.3.3 Advanced computational and imaging techniques for breast cancer detection

##### 2.3.3.1 Artificial intelligence for breast cancer detection

Since the advent of computer technology, researchers have developed automated analysis methods for medical imaging. Initially, low-level pixel processing (edge and line detector filters, region growing) and mathematical modeling (fitting lines, circles, and ellipses) were applied sequentially in medical image analysis from the 1970s to the 1990s in order to create compound rule-based systems that addressed specific tasks. Expert systems that had a lot of if-then-else statements, which were common in Artificial Intelligence (It refers to the capability of a computer to replicate human behavior, such as learning and taking action. AI developers teach computers to identify patterns in extensive datasets. After training, the program can independently analyze new data and make predictions) at the same time, can be compared to this. These expert systems, which resembled rule-based image processing systems, were frequently fragile and have been referred to as GOFAI (good old-fashioned artificial intelligence) (95). The clinical field is undergoing radical change as a result of the digital age, especially in the fields of radiology and pathology. In several fields, artificial intelligence techniques are being developed to address medical problems such diagnosis, prognosis, drug discovery, and testing (96–99). Artificial intelligence techniques have been applied specifically to breast cancer, where they have been used to diagnose (100) and prognosis, classify and quantify immunohistochemistry-stained images (101–103) and predict the pathological complete response (PCR) to neoadjuvant chemotherapy (104, 105). These applications have provided the opportunity for individualized care, increased therapy response rates, decreased adverse effects, and decreased costs of unnecessary treatment. AI has been utilized in radiology since the 1990s, initially with CADE tools in mammographic screening prompting readers to re-examine areas of concern in the image (106). With its ability to automate processes, extract minute information from photos, and provide predictive insights, artificial intelligence (AI) offers a viable solution to the problems that now exist (107–110).

In the field of diagnosis, medical images are first collected, then preprocessed, segmented, features extracted and eventually categorized. Image processing involves capturing digital images in a fixed format, usually a portable gray map. The next step is image preprocessing, which removes noise and enhances contrast using techniques like FPN, Bad pixels, temperature calibration, Vignetting, and Noise smoothing. Image segmentation divides an image into distinct sections, based on features, with the quality of the output largely reliant on measurement accuracy. Feature extraction converts input data into extracted features, such as spatial, transform, edge, color, shape, and texture features. These techniques are crucial in diagnosing disorders, particularly in distinguishing between natural and abnormal tissue features in breast masses or microcalcifications. The analysis of breast cancer sensor data has been transformed by contemporary In order to analyze and interpret the complex data produced by breast cancer detection technologies, artificial intelligence (AI) and machine learning are essential. These technologies have a number of significant benefits. Processing high-dimensional data from wearable sensors and imaging requires the ability to spot subtle anomalies and complex patterns in large datasets, which AI algorithms are better at than traditional analytical techniques. The analysis process can be streamlined by using AI to automatically extract the most relevant features from sensor data, such as subtle biomarker fluctuations or temperature variations. More precise diagnosis can be achieved by training machine learning models to categorize sensor data (e.g., differentiating between normal and abnormal tissue) and forecast the likelihood of cancer development. AI makes it possible to conduct customized analysis for each patient, taking into account their particular traits and risk factors to produce accurate and individualized evaluations. AI makes it easier to combine data from various sources, such as genetic information, imaging scans, and wearable sensors, to provide a comprehensive picture of the patient’s health. **Table 4** explains all the terminologies used in paper related to Ai and ML and how they are related to breast cancer.

**Table 4:** AI and machine learning algorithms terminologies.

Algorithm	Definition	Relevance to Breast cancer	Reference
ANN (Artificial Neural Network)	a computer model that can recognize intricate patterns and is modeled after biological neural networks.	utilized for biomarker prediction, MRI tumor segmentation, and mammography classification.	(111)
LR (logistic Regression)	A statistical model that uses logistic functions to predict binary outcomes.	uses clinical information (such as tumor size and patient age) to predict the risk of malignancy.	(112)
KNN (K-Nearest Neighbors)	Labels are assigned by a non-parametric classifier using the nearest neighbors’ majority vote.	uses similarity to labeled tumor samples to classify histopathology images.	(113)
DT (Decision Tree)	Using feature thresholds, a tree-like model divides data into branches.	finds important diagnostic characteristics, such as the shape of the tumor and calcification patterns.	(114)
NB (Naïve Bayes)	A tree-like model separates data into branches based on feature thresholds.	uses biomarker information and patient demographics to forecast the recurrence of cancer.	(115)
SVM (Support Vector Machine)	A classifier looks for hyperplanes to divide data into classes.	high precision in classifying mammograms and differentiating between benign and malignant tumors.	(116)
RF (Random Forest)	a group approach that combines several decision trees.	Feature selection for tumor subtype classification and risk prediction.	(117)
K-Means	Data is divided into *k* groups by an unsupervised clustering algorithm.	separating tumor areas with unclear borders in MRI scans.	(118)
C-Means (Fuzzy C-Means)	Data points can be assigned to several weighted clusters through clustering.	using gene expression data to stratify breast cancer subtypes.	(119)
Hierarchical clustering	creates nested clusters using agglomerative/divisive proximity matrices.	modeling intricate distributions of biomarkers to detect cancer early.	(120)
GMM (Gaussian Mixture Model)	Data are represented as mixtures of Gaussian distributions in a probabilistic model.	Analyze complex Biomarker distributions	(121)

###### 2.3.3.1.1 Machine learning algorithms for breast cancer prediction

**Figure 7:**
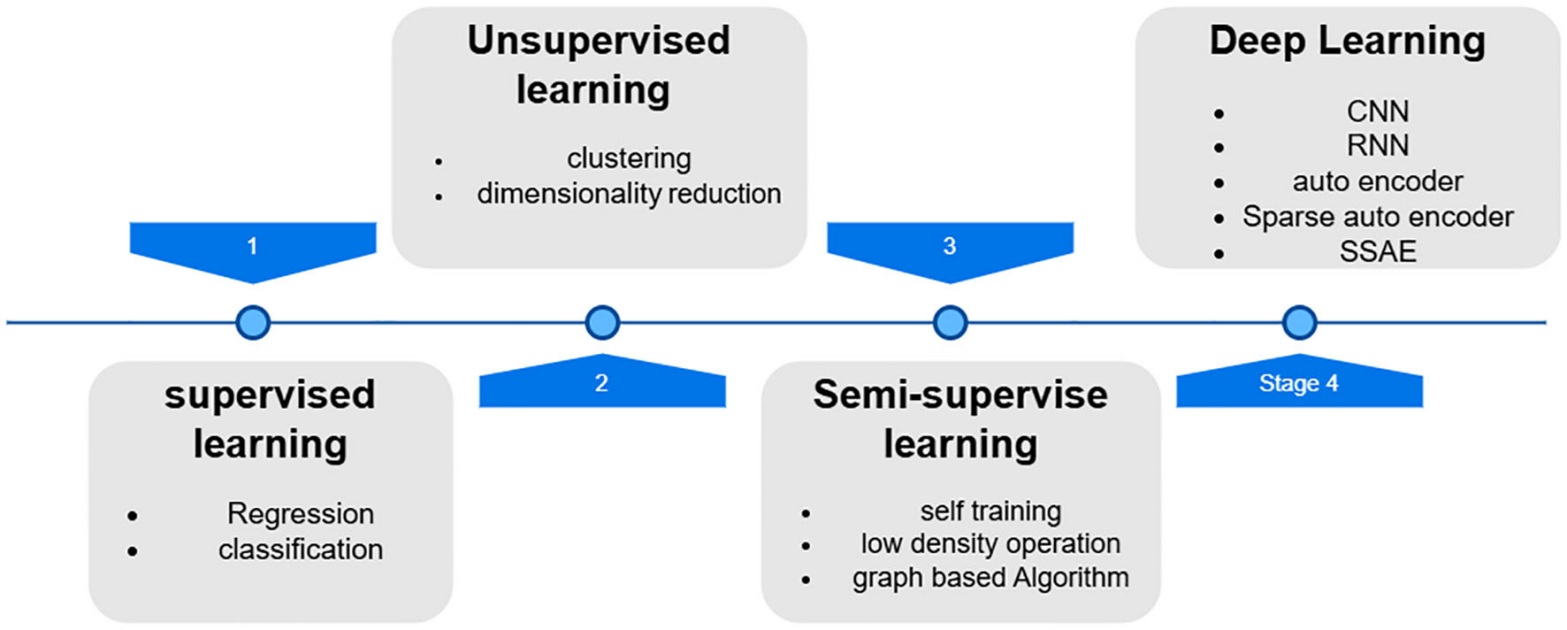
Hierarchical classification of machine learning algorithms (131).

Artificial Intelligence consists of a wide range of methods, such as machine learning, which is a subset of deep learning, of which CNNs are only one (122). Machine learning is an automated learning technique (123), with algorithms built to learn from previous datasets; we feed a mountain of data into a machine learning model, and it uses that data to anticipate what the future holds (103–106). Discuss the hierarchical classification of machine learning algorithms, which encompasses supervised, unsupervised, semi-supervised, and deep learning techniques (**Figure 7**).

Artificial Neural Network (ANN) (111) is a common data mining algorithm that consists of an input, hidden, and output layer. It is based on parallel processing (124), distributed memory (125), collective solution, and network architecture (115, 126, 127). Logistics regression (LR) (112) is a supervised learning algorithm that includes more dependent variables and provides continuous outcomes for specific data (115). K-Nearest Neighbor (KNN) (128) is used for pattern recognition and is effective for breast cancer prediction (115). Decision Tree (DT) (114) is a supervised learning algorithm that divides a dataset into smaller subsets for higher precision prediction (115). Naive Bayes Algorithm (NB) is a model used to make assumptions about a large training dataset and calculates probabilities using the Bayesian method. It is an analogy classifier that is used for comparing training datasets with training tuple (115). Support Vector Machine (SVM) is a supervised learning algorithm used for both classification and regression problems (116), providing the highest accuracy rate for large dataset predictions. Random Forest (RF) (117) is a building block of machine learning used for predicting new data based on previous datasets (115). K Mean Algorithm is a clustering algorithm that partitions data into small clusters based on similarity between data points (129). C Mean Algorithm is used for medical image segmentation and disease prediction (119). Hierarchical Algorithm evaluates raw data in the form of matrices, with each cluster separated by a probability model. Gaussian Mixture Algorithm is a popular unsupervised learning technique that computes the probability of different types of clustered data based on expectation maximization (121).

Machine learning techniques are only effective if the first input data has significant predictive characteristics. DL, a subset of ML, was created to use deep, multi-layered structures to enhance the performance of traditional ANNs. Among the various deep neural networks, CNNs rely on convolutional processes to transform unprocessed image data into intricate representations, eliminating the requirement for explicit feeding of features extracted from the image (130).

The use of AI and machine learning has led to substantial enhancements in breast cancer treatment outcomes. AI algorithms assess patient-specific data to predict treatment responses and identify the best treatment plans, thereby decreasing side effects and increasing effectiveness. AI-enhanced image analysis assists surgeons in accurately locating tumors and planning surgeries, ensuring precise tumor excision and better cosmetic results. Additionally, AI refines radiation therapy strategies for accurate tumor targeting, reducing harm to surrounding healthy tissues. Machine learning approaches evaluate patient data to forecast recurrence risk, allowing for timely interventions that boost survival rates. Notable examples include AI tools analyzing mammograms to predict breast cancer risk and customize screening schedules, as well as machine learning models that anticipate patient responses to chemotherapy. **Table 5** explains the A Comprehensive Review of Major Machine Technique from 2015-2019 (For Breast Cancer Prediction).

**Table 5:** A comprehensive review of major machine technique from 2015-2019 (for breast cancer prediction).

Technique	Description	Merits	Limitations	Year/reference
Computer-Aided Diagnosis System (CAD) for predicting breast cancer.	Involved in the comparative analysis of Machine learning algorithms including random forest, gradient boosting and K nearest neighbors was conducted.	The random forest algorithm, which combines both regression as well as classification methods, achieved the highest accuracy. It involves multiple trained models to make predictions for various training classifiers. A method called the hybrid method designed to develop accurately compute the UCI online dataset, resulting in the most and precise accurate outcomes.	The expected probabilities of result (occurrence and non-occurrence are calculated via K-fold cross-validation, which is a more expensive task. The pre-processing stage of data acquired most of the time because of raw data conversion into scalable and valuable form. Additionally, the number of patients that were already mentioned in a list was not considered.	2019 (136)
Comparison of classification algorithms through weka and spark	For the evaluation of tree types of data that contain DM, GE, and a mix of both, classification models that support vector machines, decision trees, and random forests were taken into consideration.	The support vector machine’s ability to examine several data sets simultaneously stems from its parallel computation foundation. It offers the best accuracy rate across Weka and Spark, two distinct tools. Compared to decision trees and random forests, SVM has a lower error rate and calculation time.	Gathering data on gene expression is a difficult undertaking. Many samples are needed for calculations in order to produce sensitive, accurate, and precise data.	2019 (137)
Comparison of Nonlinear Machine Learning Algorithms.	MLP in contrast to non-linear machine learning techniques like K Nearest Neighbor, Support Vector Machine, CART, and Naïve Bayes.	Because MLP is composed of multiple layers, each of which carries out a distinct task independently, the calculation of this technique was sufficiently fast. When the datasets are linearly separable, it provides a respectable level of accuracy.	The user must specify the hidden layers of the MLP algorithm. There were times when setting a value resulted in overfitting and other times in underfitting outcomes. Without 10-fold cross-validation, it is difficult to predict the accuracy rate using train data models.	2019 (138)
Comparing SVM and ANN for Breast Cancer Prediction	Metrics including accuracy, precision, recall, and ROC area were used to evaluate the performance of SVM and ANN.	Since SVM divides classes based on hyper lines and generates results with a better accuracy than ANN, it was found to be the most suitable technique for predicting breast cancer after comparison.	The predicted probability of occurrence and non-occurrence are calculated using K fold cross validation. This is a more expensive endeavor.	2019 (139)
Optimizing algorithms using genetic programming techniques	To obtain the data, digital pictures were put through feature extraction and selection processes.	Then, specific characteristics were selected and several machine techniques were compared using the polynomial features operator. The additional tree classifier yielded the highest accuracy when compared to other techniques.	The processes for training and evaluating the model were excessively lengthy. The GP algorithm was used to solve the hyperparameter problem, but processing it was extremely time-consuming.	2019 (140)
Comparative Analysis of Data Mining Classifiers for Cancer Prediction and Detection.	The classification algorithms random forest, bagging algorithm, random committee, simple CART, and IBK were investigated using k-fold cross-validation.	The adobe forest algorithm, which requires less effort, had the highest accuracy throughout the evaluation. Random forest algorithms don’t need data to be standardized or normalized, and they might be better at handling nonlinear data.	To detect malignancies, a new model was developed, however processing it took too long. Our iterative use of K-fold cross-validation led to an excessive amount of time spent on each iteration.	2019 (141)
Prediction of breast cancer using Naive Bayes, KNN, and J48.	Training data and testing data were the two categories into which the dataset was divided. Tenfold cross validation was applied to the evaluation techniques.	The most effective way to predict cancer datasets was to group the data based on the degree of similarity between each incidence. Assign high accuracy to both training and testing data.	Testing is slow and takes a long time. Choosing a K value could be difficult.	2019 (142)
Recursive Features Selection for Breast Cancer Detection	Several kernels, including linear, RBF, polynomial, and sigmoid, were used to examine the SVM algorithm. On linear kernels, SVM performed more accurately than alternative techniques.	The most accurate method for choosing relevant traits for breast cancer prediction is to use SVM linear kernels. High accuracy was achieved by developing the projected model and feature selection method for large datasets.	Calculation time arose when irrelevant information was extracted. Compared to other models, the SVM linear kernel computed more slowly and had higher error rates.	2019 (143)
Breast cancer diagnoses through classification techniques.	Using linear discriminant analysis (LND) and dimension reduction methodology, machine learning approaches are compared.	Through feature selection and extraction, the classification model—which was developed using a training dataset—improves patient classification of benign or malignant tumors while requiring less data storage	Although the evaluation step takes a long time because of CFS, LDA, and PCA approaches, the solution uses the R programming language, which has fewer packages and requires less processing than other languages.	2019 (144)
Breast Cancer risk prediction and Diagnosis.	Performance metrics evaluate the C4.5, SVM, NB, and KNN models’ sensitivity, accuracy, and precision. Out of all the models, SVM has the highest accuracy.	Each method is accurately evaluated by the ROC curve. The SVM algorithm increases the precision of accurately classifying events. The error rate value was lower for this algorithm.	The ROC curve provides an accurate evaluation of each algorithm. The SVM algorithm improves accuracy in predicting correctly classified occurrences. This algorithm had a reduced error rate value.	2018 (145)
Most effective machine learning for predicting breast cancer.	The dataset was divided in two parts. Prior to the feature extraction and selection techniques, the K-fold validation methodology was employed. SVM improved the accuracy of predictions.	SVM provided an accuracy of 99.7% for the benign class and 94.6% for the malignant class when a predictive model was developed. Compared to other algorithms, SVM has a lower error rate and a quicker turnaround time.	It’s crucial to use a suitable approach when evaluating a machine learning algorithm. The conflict matrix was created with the intended class outcome in mind; it correctively predicted the occurrences, but the maximum prediction time was used.	2018 (146)
Hyperparameter Optimization for the Prediction of Breast Cancer.	The HPO technique’s clustering method was used to identify the best prediction algorithm for breast cancer.	Hyperparameterization utilizing the clustering method yielded the highest accuracy. Hyperparameters performed better with continuous and categorical data types.	Certain features also gave some redundant data. There are too many steps in the BCOAP model, and each one takes too long to analyze breast cancer data.	2018 (147)
A Neural Artificial Network for Breast Cancer.	ANN algorithms were employed for prediction using the backpropagation process. Each hidden layer provided a different level of accuracy during evaluation.	The arbitrary weight produced by the multi-layered neural network produced the Mean Square Error, whose rate is too low. By changing the weight, the feed-forward algorithm lowers error.	Demand a lot of processing power and time for a significant amount of data, which has an impact on the data’s overall correctness. For computations, a huge number of samples are required in order to attain good accuracy, precision, and sensitivity of data.	2018 (148)
Comparing data mining techniques for the categorization of breast cancer	The fusion classifier, which combines many classifiers, was created to assess the algorithm using various data mining tools.	More accuracy was obtained from a single classification than from a fusion classification. When the confusion matrix was designed, the WPBC, WBC, and LBCD datasets offered the higher level of accuracy throughout the evaluation of various algorithms.	With the exception of the LBCD dataset, the Weka tool’s accuracy was subpar. However, it was the most accurate for the WPBC and WBC datasets.	2017 (149)
Breast cancer prediction by the use of data mining methods.	For the purpose of comparing the classification and clustering algorithms, a confusion matrix was created.	Compared to the other algorithms, the classification algorithms C4.5 and SVM produced better results. Furthermore, EM created the most effective clustering method for breast cancer.	One of the hardest tasks is figuring out the effect algorithm that predicts the onset and recurrence of diseases.	2017 (150)
Breast cancer using computer algorithms for diagnosis and prognosis.	The time build model was created to analyze the effectiveness of various classifiers.	After evaluating each classifier using a confusion matrix, SVM has a higher accuracy rate and lower error rate for breast cancer prognoses.	SVM was longer to process than KNN, however KNN was a lazy learner approach with poor accuracy results.	2016 (151)
Breast Cancer Analysis Using Classification Algorithms	The classification algorithm’s performance was evaluated using accuracy, sensitivity, and precision metrics.	We compared categorization algorithms using weighted average values. The CART algorithm accurately predicts breast cancer outcomes in a short period of time.	The model compares the data mining decision tree algorithms J48, CART, and ADtree. The evaluation step took too much time.	2016 (152)
Data mining categorization algorithms for risk prediction of breast cancer.	A performance matrix was used to compare Naive Bayes and J48 models. The J48 algorithm outperformed Naive Bayes in terms of accuracy rate.	The naive Bayes algorithm produced the lowest error rate throughout computing. Increasing the number of attributes and sample size resulted in improved accuracy.	The expression rule was intended to identify the best features for breast cancer prediction, but the review procedure was overly difficult.	2015 (153)

###### 2.3.3.1.2 Deep learning techniques for breast cancer prediction

An extension of artificial neural networks, or ANNs, is called deep learning. The architecture of deep learning algorithms is made up of numerous layers. These algorithms can recognize all of the data from various categories and are used to process a significant amount of natural data. When we have a large amount of unlabeled data, we typically use unsupervised deep learning algorithms (132). Autoencoders are neural networks that learn from large datasets by training their network to ignore irrelevant signals like noise (133, 134). Sparse auto-encoders learn from unlabeled data using a feed-forward and backpropagation algorithm, handling the sparsity regularizer (132–134). Stacked Sparse Auto Encoder (SSAE) (132) combines the basic layers to construct a stacked sparse, with hidden layers based on classifiers providing output (133, 134).

Convolutional neural networks (CNN) analyze cancer datasets using CovNet for data analysis and filters to capture different dimensions of images. CNN consists of pooling, convolutional, classification, and fully contacted layers (133, 135). Recurrent neural networks (RNNs) are a class of neural networks that consist of hidden states that use the output of previous states as input for the next state. While they can process a sequence of inputs using the same parameters at each layer, they cannot process a large number of inputs through ReLU and Tanh activation functions.

##### 2.3.3.2 Imaging techniques

**Figure 8:**
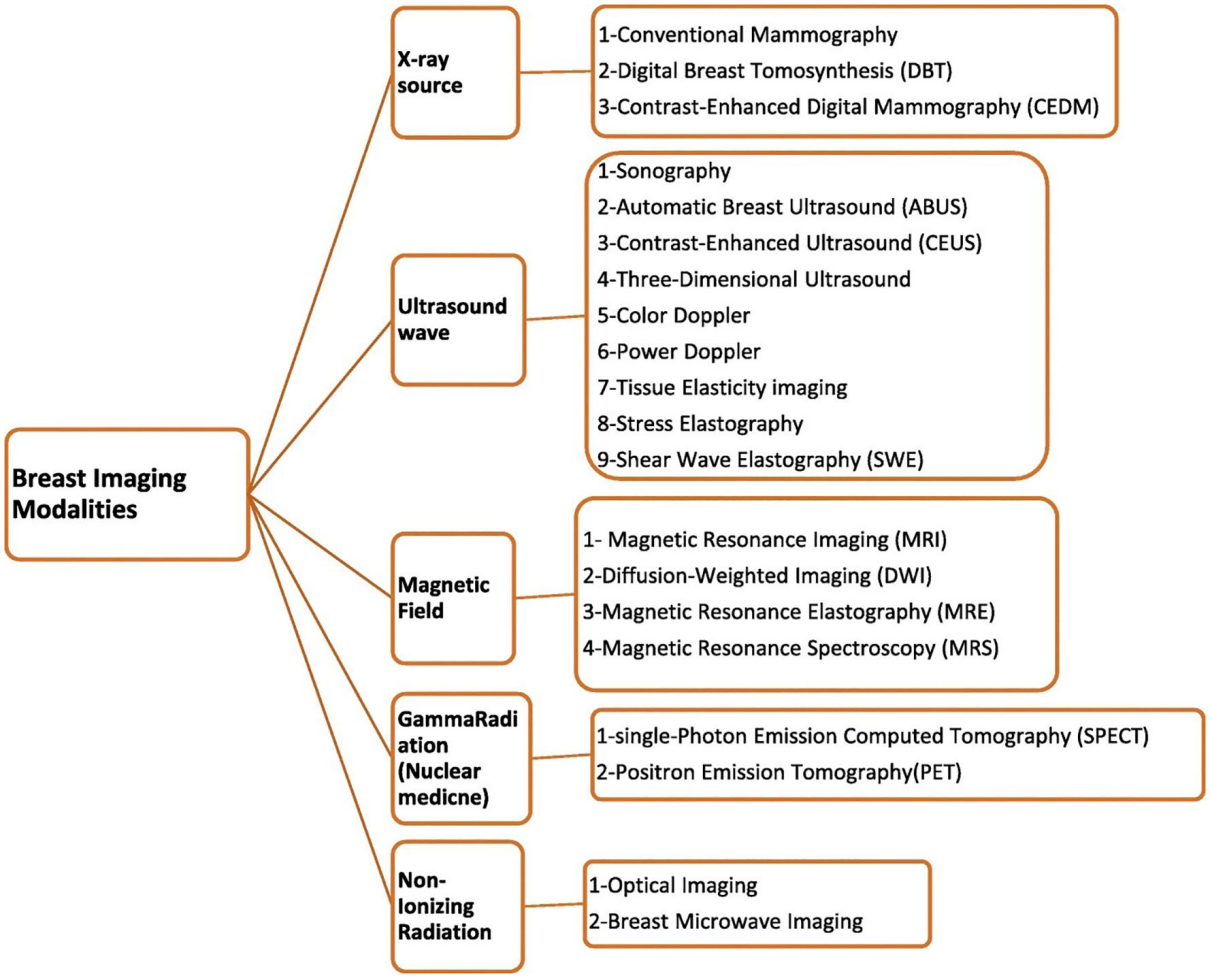
Different imaging methods in the diagnosis of breast cancer (154).

The landscape of breast cancer detection has evolved with a variety of imaging techniques, each offering its own capabilities for early diagnosis and monitoring. Artificial Neural Networks (ANN) have become an integral part of the field, applying sophisticated algorithms to analyze complex patterns in image data, thus improving diagnostic accuracy. Reflective optical imaging devices (ROIDs) provide high-resolution images by reflecting light that helps distinguish tissue types and identify potential malignancies. Microwave imaging (MI) and microwave-induced thermo acoustic imaging (MITI) represent state-of-the-art techniques that use microwave signals and thermo acoustic effects to reveal breast tissue abnormalities and provide a non-invasive method of detection. Automated Breast Ultrasound (ABUS) and Ultrasound Imaging Systems (UIS) assist the field by providing detailed, real-time images of breast tissue, making it easier to detect structural changes and abnormalities. In addition, infrared imaging technology (IIT) detects temperature fluctuations in the breast tissue, which can indicate pathological changes. Together, these imaging modalities provide a comprehensive toolkit to improve breast cancer detection, and each offers unique strengths to improve early diagnosis and treatment strategies. These all imaging techniques are explained in **Figure 8** for the diagnosis of breast cancer.

Here, we provide an overview of popular imaging modalities used in breast cancer analysis and diagnosis. Studies have demonstrated that there are various imaging modalities, such as digital breast tomosynthesis, positron emission tomography, magnetic resonance imaging, ultrasound, histopathology, mammography, and combinations of these modalities (multimodalities). **Table 6**: Summary of various imaging modalities for screening of breast cancer.

**Table 6:** Summary of imaging modalities for screening of breast cancer.

Imaging Modalities	Principles	Diagnostic accuracy	Advantages	Limitations	References
Mammography (first line tool for detecting breast cancer)	Detailed images can be found in low dose ionizing X-rays.	Sensitivity: 75–90% Specificity: 90–95% Spatial Resolution: 50 µm	most economical. Excellent reaction with a high level of sensitivity and specificity. portable gadget.	Ionizing radiation is used. As breast density increases, sensitivity falls. The accuracy of young ladies is low. Young women with thick breasts have a high rate of false-positive outcomes. In contrast to MRI, the contrast is poor.	(155–157)
Magnetic Resonance Imaging	obtains fine-grained images of the breast’s architecture using powerful magnets and low-energy radio waves.	Sensitivity: 75–100% Specificity: 83–98.4% Spatial Resolution: 25–100 µm	Capacity to identify breast cancers that frequently evade diagnosis by clinical, mammography, and ultrasound .	costly, and the test cannot be standardized. unnecessary breast biopsies as a result of the incapacity to differentiate between benign and malignant tumors.	(158–161)
Dynamic Contrast Enhanced MRI (DCE-MRI)	Several MRI scans performed after an intravenous contrast agent injection	Sensitivity: 89–99% Specificity: 37–86% Spatial Resolution: 25–100 µm	performs well in tracking reaction after treatment.	Artifacts based on tumor shape and hemorrhage caused false-negative results.	(162–164)
Diffusion-Weighted Imaging	creates contrast by using the diffusion of water molecules.	Sensitivity: 83% Specificity: 84% Spatial Resolution: 25–100 µm	Non-radioactive imaging technique	High apparent diffusion coefficients make it difficult to identify cancerous tumors with a high water content.	(165)
MR Elastography (MRE)	dynamic elasticity imaging method that blends low frequency with MRI imaging. evaluates tissue stiffness by producing an elastogram using mechanical waves.	Sensitivity: 90–100% Specificity: 37–80% Spatial Resolution: 25–100 µm	Non-invasive, non-ionizing and cross-sectional imaging modality	inability to detect small focal lesions and lack of spatial resolution.	(166, 167)
Positron Emission Tomography conjugated with computed Tomography (PET-CT)	Combines nuclear medicine technique and computed tomography resulting in high detailed images.	Sensitivity: 90–100% Specificity: 75–90% Spatial Resolution: 2–10 mm	not invasive. offers twice as many diagnostic advantages (intricate images of tissues and organs by CT scan, and elevated activity within the body detected by PET scan).	High-cost. Unable to detect tumors less than 8 mm.	(166)
Sentinel lymph node biopsy (SLNB)	Surgical procedure to detect spreading of cancer in lymphatic system.	Sensitivity: 90.5% Specificity: 85.7% Spatial Resolution: Not Applicable	Significantly reduces post-operative complications	Patients with inflammatory breast cancer and locally progressed tumors will not benefit from this treatment.	(167, 168),
Breast Specific Gamma Imaging	Employs use of a radiotracer. Image captured using a special camera.	Sensitivity: 90–96% Specificity: 71–80% Spatial Resolution: ≥7 mm	Able to identify smaller lesions (<1 cm)	High radiation dose. Not suited for routine tumor screening.	(169–171)
Ultrasound	Employs sound waves to image breast tissues	Sensitivity: 80–89% Specificity: 34–88% Spatial Resolution: 50–500 µm	Accessible, real-time lesion visualization, cost-effective, patient compliant.	Not helpful for those with inflammatory breast cancer and locally advanced tumors.	(172)

###### 2.3.3.2.1 Digital breast tomosynthesis

**Figure 9:**
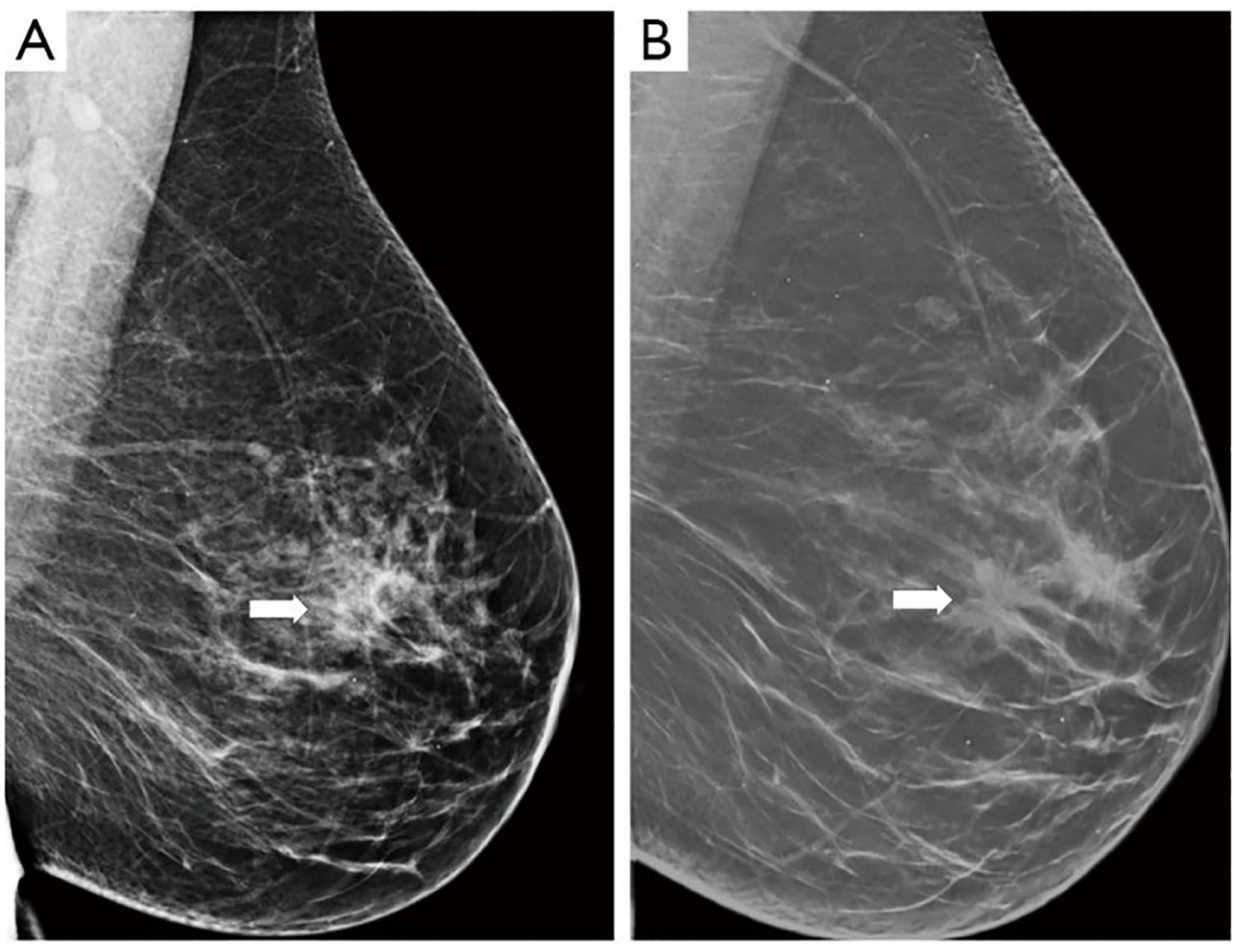
Breast Cancer Imaging: **(A)** mammography, **(B)** Digital Breast tomosynthesis (DBT) (183).

Due to the limitations of two-dimensional mammography (DM), digital breast tomosynthesis (DBT) has been developed and clinically introduced over the last two decades. DBT is an advanced imaging technique that creates 3d images of the breast and makes it easy to detect lesions and abnormalities because it reduces the chance of overlapping tissue (173). DBT detects 15-30% more cancers than mammography and reduces false positivity rate by 15-20% (174). Studies have shown that DBT increases cancer detection and can lower the recall rate depending on the baseline recall rate for DM. A review on digital breast tomosynthesis has included results of different studies on why DBT should be used in regular screenings and what its limitations are. Among the merits of dbt, it can analyze overlapping breast structures more clearly which helps radiologists distinguish normal and abnormal shadows and helps lower the number of false positive recalls (175–178). **Figure 9** clearly illustrates that Digital Breast Tomosynthesis (DBT) provides a clearer image compared to traditional mammography.

A study in 2019 aiming to compare the results of dbt over multiple years to digital mammography concluded that dbt outperformed digital mammography in detecting invasive cancers, reducing false negative rates and higher sensitivity. The findings supported the use of dbt in breast cancer screening, despite longer follow-ups and more data to support such claims (179). Another research in 2024 comparing dbt and dm (digital mammography) concluded that dbt improved cancer detection rates especially those at early stages. Findings from the paper highlight dbts potential in screening practices globally, but further long-term studies are needed to evaluate its impact on screening outcomes (180).

However, one drawback is that interpreting DBT images takes about twice as long as reading DM images due to the higher number of images. To introduce DBT into large-scale screening programs, methods to reduce reading time need to be developed. Automated interpretation methods could play a significant role in this by enabling faster image navigation and reducing variability in interpretation, potentially improving the impact of DBT on recall rate at screening. The demerits of dbt include higher radiation exposure, increased cost, longer reading times, data storage, and changes to diagnostic practice (181). Dbt also has potential for overdiagnosis which raises concern for its incorporation in daily screening (174). Although DBT has better results than mammography, it still requires extensive research to be used as a proper screening tool. This is because DBT has a higher radiation dose and longer reading time (182) **Table 7** shows the Comparison of several Digital Breast Tomosynthesis (DBT) methods, emphasizing the advantages and disadvantages of each for the identification of breast cancer.

**Table 7:** Comparison of several Digital Breast Tomosynthesis (DBT) methods, emphasizing the advantages and disadvantages of each for the identification of breast cancer.

Techniques	Advantages	Disadvantages	References
Artificial Neural Networks (ANN)	High accuracy in detecting BC Can be integrated with Computer- aided Diagnosis (CAD) systems Can be used in thermal imaging, mammography, ultrasound, MRI, and other imaging modalities.	Computationally intensive Requires large amount of data for training	(184)
Reflective Optical Imaging Device (ROID)	Offer real-time, high-resolution imaging, making potential anomalies easier to see. ROIDs are typically less expensive. More comfortable diagnostic technique to patients	Penetration limit of approximately 15 cm ROID procedures can be complex and time-consuming.	(185)
Microwave Imaging (MWI)	Non-invasive and non-ionizing Better differentiation in dense tissues Designed to be portable	Complexity of Interpretation More thorough clinical trials are required to confirm its efficacy.	(186, 187)
Automated Breast Ultrasound (ABUS)	Enhanced Visualization Automation streamlines and speeds up picture registration and segmentation.	Dependence on Fiducial Markers Limited Lesion Visibility	(188)
Ultrasound Imaging System (UIS)	Cost-Effective Non-Contact and Painless	Complex Setup Maintaining a constant temperature is crucial for accurate imaging	(188)
Microwave-Induced Thermos Acoustic Imaging (MITI)	Detects very small tumors (radius of 0.25 cm) early. Higher temperature and pressure in tumor area for distinguishability. Combines microwave and ultrasound imaging benefits.	Complex multi-physics modeling and simulations. Harder detection in glandular tissues.	(189)
Infrared Imaging Technology (IIT)	Noninvasive and radiation-free imaging. Potential for early detection of breast cancer.	Sensitivity and specificity remain less than optimal.	(190, 191)

###### 2.3.3.2.2 Reflective optical imaging device

Accurate vein identification and early breast cancer detection are critical in modern medicine. Vein location can be challenging, especially in children, obese patients, and those with difficult venous access, causing patient discomfort and complications during blood collection. Meanwhile, breast cancer remains a leading cause of cancer death globally. Traditional diagnostic methods like mammography, MRI, and CT scans have limitations such as high cost and long scan times. The BKA-06 device was developed to improve the accuracy and efficiency of detecting blood vessels and breast tumors using red to near-infrared light-emitting diodes, providing a non-invasive, cost-effective solution for real-time imaging in clinical settings (185).

The BKA-06 is an advanced medical imaging device using red to near-infrared LEDs to capture real-time images of blood vessels and breast tumors. It offers a non-invasive and cost-effective alternative for breast cancer detection, providing high-resolution images of breast tissue for early tumor detection. With a maximum light intensity of 98,592 lux, it enables thorough examinations and quick visible results. It is more affordable than MR and CT scans, making it a more accessible option for many patients. However, extensive clinical validation is needed to ensure accuracy, particularly in detecting deeper tumors. Continuous research and development are crucial to enhance its capabilities for better health outcomes and more accessible medical care.

###### 2.3.3.2.3 Microwave Imaging

MBI (microwave breast imaging system) is another imaging technique that is non-invasive, cost-effective and nonionizing which makes it safe for patients (192). Microwave Imaging (MWI) is a promising method for detecting breast cancer using non-invasive electromagnetic waves in the microwave frequency range. Tumors with higher water content than normal tissues have distinct dielectric properties that MWI can detect. Microwave antennas such as monopole antennas provide the simplest design among different antennas in MBI (microwave breast imaging systems) systems. Monopole antennas are easily fabricated into pcb, which makes it cost-effective. Slot antennas are also low-cost and offer wideband performance antennas are used in wearable systems but require improvements in radiation, bandwidth, and gain (193). Different countries have conducted research on their mbi-based prototypes. A study in UK (Bristol) claimed to achieve a sensitivity of 76% by clinical trials on 225 patients, using mbi prototype MARIA (194). Mammowave a prototype developed in Italy, gave a sensitivity of 78% in clinical trials on 58 patients (195).

The SAFE device is a noninvasive, painless, and non-invasive microwave imaging system designed for early detection of breast cancer. It uses harmless electromagnetic waves and does not require breast compression, making it a safer alternative to traditional X-rays. The device’s sensitivity varies by breast size, suggesting potential for improved detection (186, 189). Another study in the medical imaging department of Italy validated their mbi-based prototype called Wavelia on 24 subjects and achieved an accuracy of 88.5% by successfully differentiating between benign and malignant lesions (196). New technologies such as MTM (metamaterial antenna), MTS (meta surface antenna), AMC(artificial magnetic conductor antenna) are recently used by researchers. For example, the MTM microstrip patch antenna was developed in 2022 with AMC to enhance gain (197). Another MIMO (multiple input multiple output) UWB antenna was developed to improve detection accuracy to be successfully used in breast imaging devices (198). The first radar-based system was developed in 1997 for breast cancer (199). Their system was able to detect size and tumor inside the breast. Radar-based microwave imaging techniques use electromagnetic signals to create high-resolution breast images. These techniques include CMI, TSAR, MIST, MSA, and TDDA. TSAR analyzes signals that penetrate tissue, while CMI concentrates microwaves for subsurface imaging. While MSA employs several radar pairs for screening, TDDA uses algorithms to evaluate time-domain data for imaging, and MIST creates 3D images from multiple radar signals (193).

Various MWI techniques, such as microwave tomography and radar-based imaging, have shown promising results, but challenges such as variation in performance due to breast size and the need for better resolution remain. Future research will focus on overcoming these challenges and integrating machine learning to enhance MWI’s clinical applications. Overall, MWI has significant potential as a stand-alone or adjunctive tool in breast cancer screening, but further research and development are needed to fully integrate it into clinical practice (189).

###### 2.3.3.2.4 Ultrasound Imaging System

**Figure 10:**
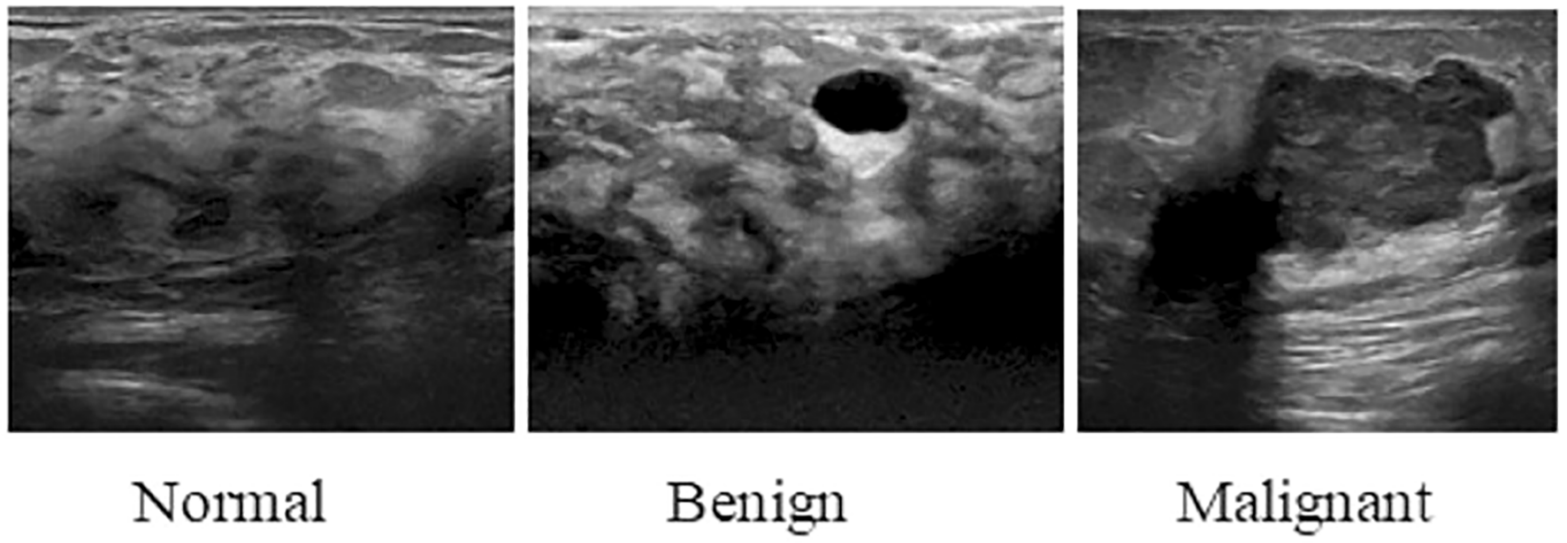
Ultrasound scans of breast in different conditions (210).

Ultrasound is becoming popular because this imaging technique is suitable for dense breasts, unlike mammography (200). Ultrasound is inexpensive and suitable for those who are not eligible for mammography. It can also prove beneficial for those who cannot tolerate breast MRI (201). Many systems have been developed for analyzing ultrasound images which are computer-aided (202). The main point highlighted in them is the need for improvement of the resolution of images (203). **Figure 10** demonstrates that Ultrasound scans of breast in different conditions. Recent studies have revealed the increased sensitivity of ultrasound for dense breasts because mammography has reduced effectiveness for that kind of breast. Mammography has the potential for false negative results, leading to the masking of abnormalities. When used as an adjunct therapy, ultrasound can identify malignancies that are often missed by mammography. This means women with dense breasts have a reduced chance of missing malignancies because of the increased sensitivity of ultrasound (22, 204–209).

Ultrasound offers real time imaging which helps in identifying minor irregularities and lesion features. This real time feature enables ultrasound guided biopsies, reducing need for more invasive procedures for breast cancer diagnosis and improved tissue sample accuracy (211–214). Recent studies suggest that ultrasound is effective for identifying tumors in young females. Because mammography is not recommended particularly for younger women and may not be as effective. Ultrasound detects lesions in a broader group of people without sacrificing its sensitivity. Thus, it allows for personalized treatment depending on every person’s risk factors (215, 216). Although there are advantages to using ultrasound as a diagnostic tool for breast cancer diagnosis it also has significant drawbacks. Ultrasound has difficulties while assessing thick breast tissues. This reduces sensitivity of ultrasound. Mammography because of its capacity to penetrate is useful while accessing thick breasts. RI offers a better diagnosis in terms of thick breast tissue (206, 217). Ultrasound is operator-dependent, and its results depend on the skills of operator compared to MRI and mammography which are regarded as more objective and do not rely on operator for the interpretation of images (218–220). Ultrasound has the ability to be used as independent screening tool for breasts, but More research and rigorous clinical trials are needed to assess the efficacy and limitations of utilizing it as the primary screening method (221). Ultrasound is dependent on the operator. To overcome this problem automated breast ultrasounds can give more fruitful results (222).

####### 2.3.3.2.4.1 The inherent limitations and biases of particular imaging techniques

Although imaging methods are essential for detecting breast cancer, it is important to recognize their inherent drawbacks and possible biases. Ionizing radiation is used in mammography, for instance, and although the dosage is usually low, repeated exposure over time, especially during long-term follow-up, increases the risk of radiation-induced cancer. Furthermore, because tumors and dense tissue can appear similar on mammograms, abnormalities may be obscured, reducing the sensitivity of mammography in women with dense breast tissue. Some women may be discouraged from getting screened for breast cancer on a regular basis due to the discomfort of breast compression during the procedure. Another popular imaging technique, ultrasound, has limited specificity and is operator-dependent, which means that different people may interpret it differently. Additional biopsies are often necessary to distinguish between benign and malignant lesions. Although magnetic resonance imaging (MRI) provides good soft tissue contrast, it is more costly and less widely available than other methods. Additionally, it has a higher risk of false positives, which could result in needless procedures. Additionally, patients who have certain metallic implants should not have an MRI. Lastly, compared to mammography, other imaging methods such as CT and PET scans require much larger doses of ionizing radiation, which raises concerns about radiation exposure. Additionally, PET scans may have limited spatial resolution. Digital breast tomosynthesis (DBT), contrast-enhanced mammography, and the creation of artificial intelligence (AI) algorithms to help with image interpretation and boost diagnostic accuracy are some of the innovations being researched to help overcome these constraints.

#### 2.3.4 Sensors

Sensors offer painless and non-invasive diagnosis of breast cancer (223, 224). In comparison to other modalities, it reduces safety threats, allowing women to receive routine breast cancer screening (225). The work done on sensors and sensor-based devices between 2015 and 2024 is explained in the following section.

**Table 8** outlines the sensors used in experimental configurations, including the models of the sensors, how they are oriented during testing, and the technology used to identify breast abnormalities.

**Table 8:** An outline of the sensors used in experimental configurations.

Name of sensor	Model	Orientation of sensors in experiment	Name of technology used	Reference	Pictures of devices:
Digital temperature sensor	ADT7420	Biometric patch	Thermography	(239)	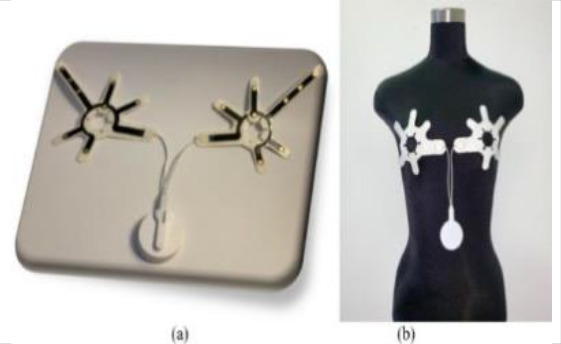
Piezoresistive sensor	Not mentioned	Vertically/perpendicularly	Not mentioned	(231)	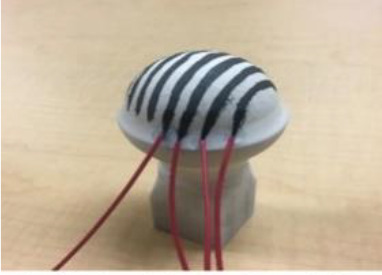
IR theomorphic sensor	FLIR A 300	Not mentioned	Thermography	(243)	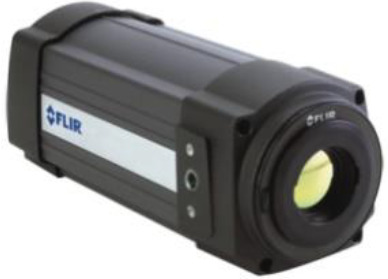
IR imaging sensor	AMG 8833	Sensor embedded in bra cup	Thermography	(229)	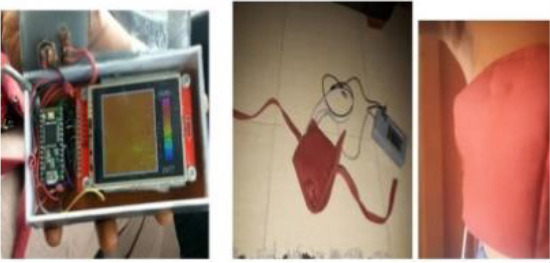
Multiple leads	Not mentioned	In form of array	NIRS	(244)	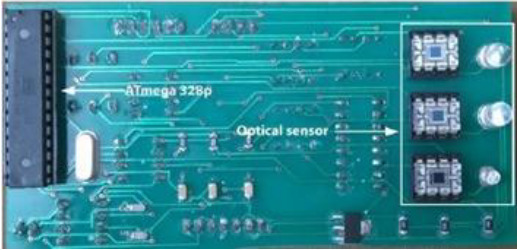
Bioimpedance sensor	Not mentioned	Not mentioned	BIS	(244)	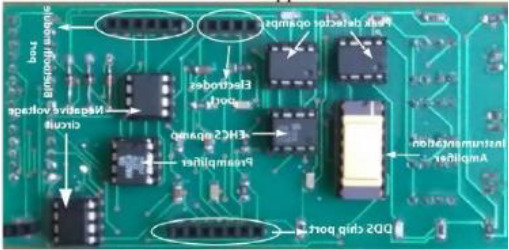
Thermal Sensor	LM35	In quarterly order	Thermography	(228)	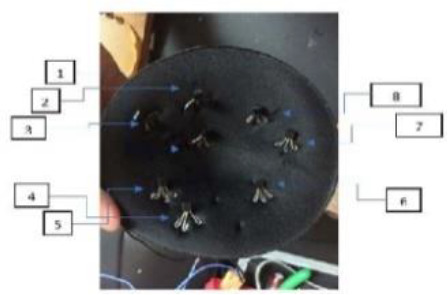
Antenna sensor	Not mentioned	Circular/Rectangular	Microwaves	(242)	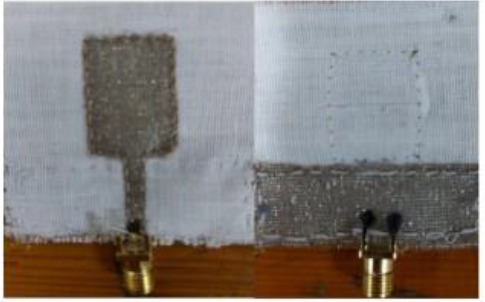
**Textile sensor**	Not mentioned	Circular	Microwaves	(225)	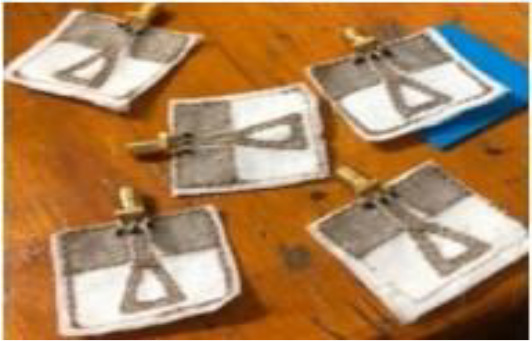

##### 2.3.4.1 Thermography based sensors

**Figure 11:**
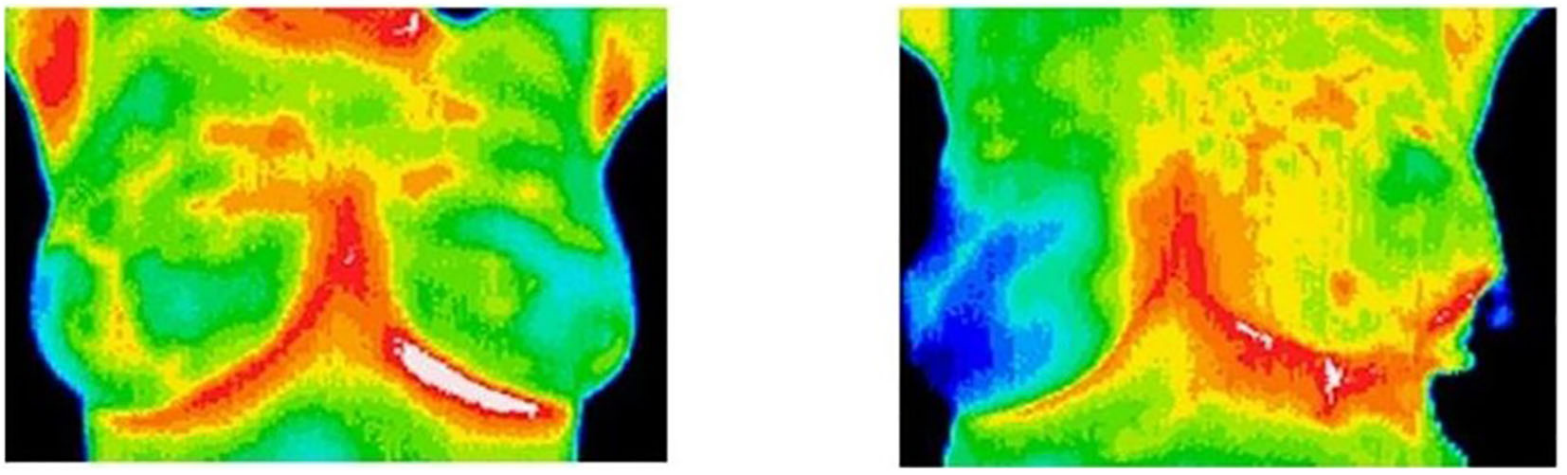
Normal vs abnormal breast thermogram.

Thermography is a commonly used method for the detection of Breast Cancer. In essence, thermography uses imaging technologies such as cameras and sensors to map the variations in breast temperature. The idea behind this procedure is that when the breasts experience abnormalities, the blood flow pattern to them is altered, which causes significant temperature variations (226). When using the current detection technologies, such as mammography, women with higher breast density levels frequently receive the wrong diagnosis. For women with higher breast densities, thermography is a useful approach (227). **Figure 11** demonstrates the Normal vs Abnormal breast thermogram.

Sensors integrated with breast thermography make an excellent combination because they are small, cost effective and easily available. A 2018 study created a Breast Cancer detecting device by correlating the output from a thermographic camera and a thermal sensor. According to the study, the thermal sensor and camera produced almost identical results, suggesting that this gadget could be useful for BC screening. The sensor LM35, FLIR C2 thermal camera, microcontroller, heater (which was imitating a tumor), and breast phantom were the materials used. On the bra pad, eight heat sensors were arranged quarterly (228).

Many studies have been carried out in which sensors based on the concept of thermography are being attached to some kind of patches or brassiere with proper skin contact (227). For example, a 2020 study that used infrared thermography to create a smart bra. Irt sensor AMG8833, microprocessor, and image acquisition system were among the materials employed. By gathering radiation from the breast region, the infrared sensor was able to produce a thermal gram based on variations in temperature. The sensors were created for both the left and right breast. When conducting self-examinations at home, this can be more helpful than potentially dangerous alternative diagnostic techniques (229). Another study from the same year used thermal microsensors as a 3*3 matrix. CMS sensors, a PCB with embedded microsensors, a processing unit, a Raspberry Pi web server, and a Wi-Fi system for temperature data transmission made up the system. CMS sensors have an operational temperature range of 0 to 50 degrees Celsius and are small, sensitive, and have low energy consumption. The future aim of the paper was to increase the number of microsensors in order to improve the quality of thermal photographs (230).

###### 2.3.4.1.1 Piezoresistive sensors

A critical component of early cancer detection is clinical breast examination. When a Piezoresistive sensor comes into contact with a lump, it changes its electrical resistance. In 2020, an automated probe that can take the role of CBE was introduced. This probe made use of a microprocessor and Piezoresistive sensor based on electro graphite. According to research on this idea, which is allegedly still ongoing, the probe can detect the position, depth, and size of the mass but is unable to distinguish between normal and pathological breast masses. Future developments in this field will focus on improving sensor calibration, streamlining the production process, and creating real-time smartphone applications (231).

In another study, a piezoresistive sensor was used in an Intelligent Breast Exam device (iBE) to detect breast stiffness. This non-invasive, low-cost method was used for screening. The device was part of a comparative study with clinical breast exams and mammography. While iBE and CBE missed some small lesions, they can be utilized in resource-limited areas for early detection (232). In 2021, a piezoresistive fabric sensor was utilized to enhance clinical breast exam simulators. The sensor performance was compared to existing sensor maps of the simulator. The sensors did not interfere with the clinical examination and also improved the measurement capabilities of the simulator (233).

##### 2.3.4.2 Near infrared and bio-impedance spectroscopy-based sensors

Another radiation-free near-infrared sensor was utilized in a paper published in 2016. The beer lambert theory forms the basis of this tumor detection technology. An array sensor produces light in the infrared spectrum. Technology can distinguish between a healthy breast and an unhealthy breast based on the amount of light absorbed by breast tissue. The development of breast phantom models with various scattering and absorption coefficients was the main goal of the project. For the others, the breast with the normal absorption coefficient and representation was used as a guide. Breast phantoms were made with India ink to resemble tumors. The suggested method of determining the ink concentration in sick breasts worked well (234). In 2018 near-infrared spectroscopy and bioimpedance-based sensors were utilized in the same device. Bioimpedance has been used to differentiate healthy tissues from cancerous tissues (235). The control unit received the results from both sensors. In recent years bioimpedance is increasingly used in bioengineering and many products have been launched in the market (236). The PCB was used to implement the entire device. The gadget and app were connected via a Bluetooth module as well. BIS intended to measure output impedance after stimulating breast tumors with low current. In contrast, NIRS sought to employ various LEDs with various wavelengths. The photo detector OPT101 is used to measure the NIRS output. The suggested device is known to have a 99.3% accuracy rate in addition to its 80mW low power consumption (237). Bioimpedance spectroscopy has a bright future in terms of human health applications (238).

##### 2.3.4.3 Digital temperature sensors

In 2020, a circadian device with digital temperature sensors was created with the goal of enhancing breast cancer detection in conjunction with other modalities. Temperature sensors ADT7420 were integrated into patches designed for both breasts, with eight sensors per patch. For a full day, the device recorded data. This sensor-based artificial intelligence device was claimed to have a 78% accuracy rate in differentiating between malignant and benign tumors. The device’s accuracy is similar to that of mammography. However, some temperature data values were missing as a result of the sensor’s inadequate skin contact (239).

##### 2.3.4.4 Microwave based sensors

Diagnostic techniques like mammography, x-rays, ultrasounds, and CT scans have been used for ages to identify cancer early still more advanced techniques are required (193). It has been demonstrated that recent developments in microwave imaging and sensing is offering ease in the detection of breast cancer. The promising results of using antennas to deliver microwaves have attracted the attention of numerous researchers. Antenna has shapes from simple to spirals (240). An attempt was made in 2016 to incorporate a microwave-based antenna array for screening purposes in a bra (241). It has also been demonstrated that circularly polarized microwave sensors, which have an axial ratio that makes them more efficient than linearly polarized ones, are safe to employ in brassieres (225). The addition of microwaves to an imaging system can improve its capabilities for breast cancer diagnosis. A study in 2016 with a compact micro strip antenna proved to be sensitive and effective on detecting breast tumors when the antenna was in contact with the skin. This antenna could easily be implemented in UWB microwave imaging system for more enhanced diagnosis. The experimentation on textile monopole sensors in 2021 did simulations of rectangle shaped monopoles with and without tumor (242). A more enhanced version of this work came out in 2023, where rectangle and circular antennas were used. Computer simulations as well as experimentation on breast models were conducted. The study claims antennas to be effective on tumors greater than 5mm only (225, 242). If we are considering using microwave imaging for diagnosis, we should employ sensor arrays while working with microwave sensors, and we should also examine their sensitivity since this will directly affect the quality of the images. Uneven spacing between sensor arrays and approaches like compressed sensing in signal and image processing should be taken into consideration if image quality improvement is to be achieved at a reasonable cost (224).

#### 2.3.5 Wearable technology for breast cancer detection

Novel, non-invasive techniques for the early detection of breast cancer have been made possible by recent developments in wearable technology. New developments in wearable technology provide a continuous, non-invasive, and patient-friendly method of screening for breast cancer. In order to identify physiological and molecular alterations suggestive of cancer, these devices combine biosensors, imaging elements, and artificial intelligence (AI) algorithms. With an emphasis on smart bras and other cutting-edge gadgets, this section examines the creation, clinical validation, engineering difficulties, and socioeconomic effects of wearable technologies. These devices are a promising replacement or addition to conventional diagnostic methods like mammography and ultrasound because they provide real-time data analysis, enhanced comfort, and continuous monitoring.

Wearable technology uses biosensors to continuously track physiological biomarkers like blood flow, tissue elasticity, electrical impedance, and temperature. Among these, smart bras with optical, piezoresistive, and electrochemical sensors have shown encouraging outcomes in identifying abnormalities in breast tissue early on. These tools have the potential to increase early detection, especially in women with dense breast tissue, which lowers the sensitivity of mammography.

##### 2.3.5.1 Working principles of wearable technologies

Wearable devices incorporate sophisticated engineering principles to facilitate continuous, non- invasive monitoring of physiological and biochemical signals. Below, we detail their fundamental operational mechanisms:

###### 2.3.5.1.1 Flexible electronics and sensor integration

Contemporary wearables utilize flexible materials (such as polymers, graphene, or elastomers) to adapt to the skin’ s contours, promoting comfort and ensuring signal accuracy. These materials house microsensors (like strain gauges and temperature sensors) and biosensors (such as electrochemical and optical sensors) to monitor biomarkers (like glucose, lactate, or pH) and physiological parameters (such as heart rate or movement). For instance, stretchable circuits printed on polydimethylsiloxane (PDMS) allow for real- time tracking of joint movements during rehabilitation (245).

###### 2.3.5.1.2 Biosensing mechanisms

Optical Biosensors: Leverage light- matter interactions (e.g., photoplethysmography (PPG) used in smartwatches) to gauge blood oxygen levels (SpO _2_) or pulse rates. They also use techniques such as surface plasmon resonance (SPR) and near- infrared spectroscopy (NIRS) for biomarker identification.

Electrochemical Biosensors: Identify analytes through redox reactions (for example, glucose oxidase- based sensors utilized in diabetes management patches). These systems typically employ enzyme- coated electrodes to transform biochemical signals into electrical outputs.

###### 2.3.5.1.3 Energy harvesting and power management

Wearables adopt energy- efficient designs to enhance their operational lifespan:

**Battery- Powered Systems:** Most commercial devices, such as fitness trackers, are powered by miniaturized lithium- ion batteries.

**Energy Harvesting:** Innovative solutions like triboelectric nanogenerators (which convert mechanical energy from movement) and solar cells (which harness light energy) aim to create self- sustaining devices.

###### 2.3.5.1.4 Wireless data transmission

Low- power transmission protocols, including Bluetooth Low Energy (BLE) or ZigBee, facilitate the transfer of data to smartphones or cloud services. Edge computing minimizes latency by processing data locally before it is sent.

###### 2.3.5.1.5 Signal processing and machine learning

Raw sensor data undergo filtering (for instance, noise reduction through Kalman filters) and are analyzed with algorithms (like neural networks) to derive actionable insights (such as detecting arrhythmias from ECG signals).

##### 2.3.5.2 Smart bras

A leading wearable technology for breast cancer detection is the smart bra. These bras are equipped with various biosensors that monitor physiological indicators such as blood flow, tissue stiffness, and temperature, each potentially signaling the early stages of tumor development. By employing IoT-enabled sensors, including ultrasound transducers and thermal sensors, smart bras facilitate early detection by consistently observing breast tissue. They can identify irregularities like temperature fluctuations, changes in density, or concerning lumps (246).

###### 2.3.5.2.1 Thermosensors

Because tumors typically have higher metabolic activity, heat is produced locally. Thermal sensor-equipped smart bras, like Cyrcadia Health’s iTBra, continuously measure the temperature of the breast skin to identify anomalies. Clinical studies showed sensitivity rates that were on par with mammography, especially in dense breast tissue where conventional imaging is ineffective.

###### 2.3.5.2.2 Piezoresistive sensors

These sensors identify variations in the elasticity of breast tissue, which may indicate the existence of tumors. Research has indicated that modifications in mechanical characteristics frequently precede detectable morphological changes.

A pilot clinical study evaluating a thermal-based smart bra reported promising sensitivity in detecting asymmetric heat patterns associated with malignancies, supporting its potential in early detection strategies.

##### 2.3.5.3 Wearable ultrasound patch

The creation of a wearable ultrasound patch by Massachusetts Institute of Technology (MIT) researchers is a noteworthy development in wearable technology for breast cancer. This cutting-edge tool allows users to perform breast imaging at home by attaching to a bra. This patch’s technology uses a miniature ultrasound scanner that creates high-resolution images of breast tissue using piezoelectric materials. Users can obtain a level of detail similar to that of conventional ultrasound probes used in medical imaging centers by moving a tracker along the patch to image the breast from various perspectives (247). This scanner’s initial tests have shown that it can identify cysts as small as 0.3 cm in diameter, which is in line with early-stage tumors.4. By enabling more frequent screening, especially for high-risk individuals who may develop cancer in between routine mammograms, this capability holds great promise for increasing the overall survival rate for patients with breast cancer (247). Additionally, this technology may make breast cancer screening more accessible in rural areas or in less developed nations. Even though there are still efforts to reduce the size of the imaging system and integrate artificial intelligence for image analysis, the device is a big step in the direction of more accessible and individualized breast cancer detection.

##### 2.3.5.4 Biosensors for biomarker detection

Wearable biosensors target specific biomarkers present in bodily fluids, such as blood, sweat, or interstitial fluid, providing early biochemical evidence of malignancy.

###### 2.3.5.4.1 Photonic Crystal Fiber Surface Plasmon Resonance biosensors

PCF-SPR biosensors are highly sensitive to molecular interactions, making them ideal for detecting breast cancer biomarkers like HER2 and CA15-3. Recent advancements in PCF-SPR technology have demonstrated high sensitivity and selectivity for breast cancer detection at low biomarker concentrations. Another study explored SPR biosensors for early cancer detection, noting their potential for non-invasive and real-time monitoring.

###### 2.3.5.4.2 Electrochemical and optical biosensors

These devices measure electrical and optical signals when interacting with specific cancer biomarkers. They offer the advantage of being cost-effective and suitable for at-home use.

Additionally, scientists are working to create customized wearable technology that uses biosensors to analyze bodily fluids non-invasively to find cancer biomarkers. These cutting-edge gadgets, which make use of thin and flexible sensor arrays, can be made into a variety of form factors, including contact lenses, wristbands, mouthguards, and headbands (248). These wearables with biosensors are primarily designed to allow for continuous and real-time monitoring of molecular markers that may indicate the occurrence or recurrence of breast cancer. Early detection, treatment efficacy monitoring, and even the identification of interval cancers—cancers that might arise in between planned screenings—are all possible with this strategy (248). Even though this technology is still mostly in the early stages of development, it is a major step forward for non-invasive diagnostics and could lessen the need for frequent, invasive procedures.

##### 2.3.5.5 Wearables for real-time imaging

Continuous breast tissue monitoring is now possible without the need for expensive imaging facilities thanks to advancements in wearable technology.

###### 2.3.5.5.1 Microwave Imaging

Wearable MWI devices identify variations in breast tissue’s dielectric characteristics. Because malignant tissues contain more water, they can be identified.

###### 2.3.5.5.2 DICOM analysis integration

Continuous, high-resolution imaging and automated anomaly detection outside of clinical settings are made possible by DICOM (Digital Imaging and Communications in Medicine) analysis, which is transforming wearable technologies for breast cancer detection. Its incorporation into smart bras and chest-worn devices, which have historically been used for hospital imaging, offers real-time monitoring, early detection, and enhanced accuracy, especially in dense breast tissues. These devices leverage machine learning algorithms to classify lesions and transmit data remotely, enhancing accessibility through telehealth platforms. Recent studies, such as those employing DICOM-based CT chest imaging (249), show comparable sensitivity to conventional methods with better patient compliance. However, challenges in data management, privacy, and regulatory approval remain. These obstacles should be overcome by developments in edge computing and secure data protocols, making DICOM-integrated wearables a game-changing instrument in breast cancer screening.

##### 2.3.5.6 Artificial intelligence in wearable technologies

###### 2.3.5.6.1 Convolutional Neural Networks

CNNs are used in smart bras and other wearable devices to distinguish between benign and malignant tissue patterns; these models increase sensitivity while lowering false positives.

###### 2.3.5.6.2 Machine learning for risk stratification

Machine learning algorithms evaluate patient-specific data, such as age, genetic factors, and hormonal profiles, to predict breast cancer risk and recommend customized screening intervals.

####### 2.3.3.6.2.1 Challenges in integrating AI with biosensors and wearable technologies

There are many obstacles to overcome when integrating AI-powered imaging with data from wearables and biosensors, especially when it comes to data privacy, effective data transfer, and the requirement for real-time, on-the-fly processing. The complexity is increased by the need for complex fusion algorithms due to the varied nature of the data produced by these technologies (such as photos and time-series sensor readings) (250). Since biosensors and wearable technology gather private health data, data privacy is crucial. Strong encryption and access control systems are essential for ensuring compliance with laws like HIPAA (in the US) and GDPR (in Europe) (251). Another challenge is the effective transfer of data from biosensors and wearable technology to processing units. High-bandwidth communication channels and optimized data compression techniques are necessary to prevent bottlenecks and delays due to the sheer volume of data, particularly when combined with imaging data (252). Additionally, real-time applications like instant feedback or alerts frequently call for on-the-fly processing. This calls for strong computer resources and effective algorithms that can process and analyze data almost instantly, which is computationally demanding and difficult for wearable technology with constrained processing power (253).

####### 2.3.3.6.2.2 Challenges in implementing wearable technologies

Wearable technologies designed for breast cancer detection show great potential, but several hurdles need to be overcome to achieve widespread use in clinical settings.

**Accuracy and reliability:** While advancements in technology are promising, the issues of false positives and false negatives persist. Over-detection can cause unnecessary worry and lead to invasive procedures, whereas missed diagnoses may delay crucial treatment. To enhance diagnostic accuracy, ongoing sensor calibration and the creation of sophisticated signal processing algorithms are vital.

**Battery life and material durability**: For continuous, long-term monitoring, devices must be made of robust materials with efficient power management. Products like smart bras and biosensors need to perform reliably even after extended use. Researchers are investigating flexible electronics and energy-efficient circuits to improve both battery life and the durability of materials.

**Cost and accessibility:** The steep production costs and the complexity of advanced sensor technology can make these wearable devices too expensive for many, especially in low-resource environments. Strategies like government subsidies, collaborations with healthcare organizations, and scalable manufacturing processes are essential for lowering costs and advancing global adoption.

Tackling these challenges is essential for wearable technologies to evolve from experimental tools into dependable clinical instruments, significantly enhancing early breast cancer detection and improving patient outcomes.

Accessibility and affordability are key factors in the broad adoption of wearable breast cancer detection technologies. These devices could become more economically feasible for mass production if production costs are lowered by developments in flexible electronics, material science, and 3D printing (254). Government policies and non-governmental organization (NGO) initiatives are also essential in bridging the gap between public health needs and technological innovation. These life-saving technologies can reach low-income populations with the help of subsidies and healthcare funding, especially in settings with limited resources where traditional screening methods are less accessible. Additionally, by enabling remote screening and follow-up care, the combination of wearable technology and telehealth services offers a revolutionary approach that lowers logistical and geographic barriers to healthcare. Wearable technology holds the key to solving infrastructure and economic issues.

Wearable technology has a lot of promises for detecting breast cancer, but there are still a number of practical issues. Because false positives or negatives might cause needless concern or missed diagnosis, accuracy and dependability are critical issues. Long-term use also depends on battery life and material durability, particularly for devices that are intended for continuous monitoring. Widespread accessibility is nevertheless hampered by high expenses, especially in environments with limited resources. Beyond technical constraints, moral considerations are becoming more and more important. These include preventing AI biases that could impair the accuracy of diagnoses in a variety of populations, guaranteeing informed consent, and protecting private patient data gathered by wearables. To achieve fair, secure, and clinically successful deployment, addressing these issues calls for strong data governance frameworks, inclusive algorithm training, and supportive public health policies.

**Table 9** shows the Weaknesses of Wearable Technologies for Breast Cancer Detection and Potential Solutions.

**Table 9:** Weaknesses of wearable technologies for breast cancer detection and potential solutions.

Weakness	Description	Impact on Detection	Potential Solutions	References
False Positives/Negatives	The presence (false positive) or absence (false negative) of breast cancer may be misidentified by wearable technology.	False positive results lead to worry, pointless testing, and higher expenses. False negative results can worsen outcomes by delaying diagnosis and treatment.	Enhanced sensitivity and specificity of the sensor. sophisticated algorithms to distinguish between signals that are benign and those that are malignant.	(255)
Reliability of Continuous Monitoring	Wearable sensor accuracy can change over time and be impacted by motion artifacts, skin contact, and sensor drift.	Inaccurate or inconsistent data limits the device’s clinical utility and undermines confidence in its readings.	methods for calibrating sensors to account for drift. sturdy designs to reduce the effects of environmental influences and motion.	(256, 257)
Clinical Utility	Wearable technology’s place in standard clinical practice is still developing, and there are still issues with data interpretation and integration.	ambiguity regarding the incorporation of wearable data into decisions regarding diagnosis and treatment. absence of established procedures for interpreting data.	extensive clinical studies to confirm efficacy. Rules for clinical decision-making and data interpretation.	(258, 259)
Data Interpretation	It can be challenging to distinguish between signs of cancer and normal physiological changes; multimodal analysis and sophisticated algorithms are needed.	may lead to either a failure to recognize actual problems or an overabundance of data analysis and unnecessary actions.	Machine learning algorithms uncover subtle patterns that could be signs of cancer. A more comprehensive analysis can be accomplished by combining different data streams.	(260, 261)

####### 2.3.3.6.2.3 Wearable technology’s cost-benefit analysis for breast cancer screening

To assess the clinical and financial feasibility of these novel methods, a thorough cost-benefit analysis contrasting wearable technology with traditional breast cancer screening is necessary. Both direct costs, like the equipment, personnel and Maintenance, and indirect costs, like lost productivity and travel/logistics, must be taken into account in this analysis. Furthermore, it is important to carefully evaluate how wearable technology might enhance scalability and lessen the strain on healthcare systems. This section offers a preliminary summary of the main cost and benefit factors involved, but a definitive analysis necessitates more investigation and long-term data. To qualitatively assess traditional breast cancer screening techniques, such as mammography and MRI, against emerging wearable technology methods, the authors created a conceptual cost-benefit framework illustrated in **Table 10**. Conventional screening techniques usually entail significant initial equipment and facility expenses, alongside ongoing costs for clinical appointments, technician labor, and maintenance. Conversely, while still in clinical development, wearable devices have the potential to lower operational costs due to continuous home monitoring and a diminished necessity for frequent hospital visits. Our analysis indicates that the long-term costs of wearable screening might be more advantageous, particularly in minimizing expenses related to delayed diagnoses and treatments. The authors’ analytical review of cost estimates, clinical workflow needs, and diagnostic features from diverse academic literature, healthcare technology assessments, and market analysis served as the foundation for the data presented, which was not derived from a single empirical source. It’s essential to note that most wearable breast cancer detection technologies remain in pre-commercial or experimental phases and have yet to secure full clinical approval for routine diagnostic use. Therefore, this comparison aims to explore how wearable systems may complement traditional modalities, highlighting possible benefits and drawbacks. The figures provided do not indicate precise financial modeling or direct clinical equivalency; they are instead illustrative, intended to facilitate forward-thinking discussions about cost-effectiveness, patient experience, and healthcare access **Table 10**.

**Table 10:** Cost-benefit analysis framework for breast cancer screening methods.

Cost/Benefit Category	Conventional Screening (e.g., Mammography)	Wearable Technology-Based Screening
Direct Costs		
Equipment	MRI, Mammography machines are highly costly(approx; mammography: $ 150,000 – $500,000, MRI: $1,000,000 – $3,000,000+)	Less than the cost of an MRI, mammography
Personnel	Radiologists, technicians. or any staff member required	Minimal staff required, mostly automated
Maintenance	10,000-50,000/year for machine maintenance	It’s less than the conventional, we have to check the software and sensors just
Indirect Costs		
Lost Productivity	Workdays missed for appointments	Home-based monitoring
Travel/logistics	Transportation cost if a person travels for screening, and this also includes the cost for the transporting machines to rural areas etc	none
Benefits		
Early detection	Detect tumors mostly less than 5mm (85% sensitivity)	It can detect early because of biomarker based detection
Patients comfort	Moderate; if a person is claustrophobic in MRI or the pain during compression in Mammography	High; radiation free
Cost savings	If detected earlier, than saves the cost of late treatment	It can give us long term savings for long-term real-time monitoring
Challenges		
False positive	~10% false positives	This is an emerging technology so it has a higher risk of false positives
Accessibility	In a low-resource region, it is difficult for the availability of such equipment	Requires internet, data security
Regulatory hurdles	Approvals from FDA	Its novel device so requires more approval

Wearable technology has the potential to change cost distributions, as shown by the data in Table. For instance, frequent clinical visits may result in lower costs, but it is necessary to account for the cost of wearable technology and the infrastructure that supports it. Furthermore, even though they are hard to measure in monetary terms, wearable technology’s increased convenience and decreased patient anxiety represent substantial potential advantages that ought to be taken into account in addition to the financial considerations. Opportunities for long-term cost savings and better health outcomes are also presented by wearable technologies’ scalability, particularly in remote areas, and their potential for personalized screening. To provide a more conclusive cost-benefit analysis, however, more research is required, including extensive clinical trials and health economic studies.

#### 2.3.6 Smart implants in breast cancer detection

Smart implants are cutting-edge, miniaturized devices specifically engineered to be seamlessly implanted within breast tissue, where they play a crucial role in monitoring vital physiological changes and identifying biomarkers that may signal the early onset of cancer. Smart implants employ microfluidic channels and biosensing electrodes to measure biomarker concentrations in interstitial fluids. When they identify cancer-specific biomarkers, such as HER2 or estrogen receptors, these implants wirelessly transmit data to external monitors for analysis. This continuous real-time monitoring system serves as an early warning mechanism for tumor progression.

Now we will explain the smart implants working in the following workflow:

➢ **Integrated sensing technology**

These sophisticated implants are equipped with advanced sensors that continuously gather real-time data on various parameters, such as temperature fluctuations, pH levels, and other biochemical markers that can indicate cancerous activity (262). Implanted microsensors identify physical alterations (such as tissue density or impedance) or tumor-associated biomarkers (such as pH changes, hypoxia, or HER2 proteins) connected to the advancement of cancer (263).

➢ **Wireless data transmission**

The information collected by the implants is transmitted wirelessly to external devices—like smartphones or specialized monitoring systems enabling healthcare professionals to analyze the data with remarkable accuracy. This innovative approach not only allows for the continuous assessment of changes within the body but also acts as a proactive health management tool.

• **Power Management and Miniaturization:**

Smart implants are engineered with advanced energy-efficient components that prioritize long-term viability. Some utilize innovative wireless energy harvesting technologies, drawing power from ambient sources or employing cutting-edge miniature battery systems. This thoughtful integration allows for prolonged functionality within the human body, significantly reducing the need for surgical replacements or interventions, thereby lessening the burden on patients and healthcare systems alike.

**Clinical Relevance:**

These smart implants facilitate continuous, high-precision monitoring of breast tissue, employing sophisticated sensors to detect minute changes in tissue composition and density. This capability enhances the potential for early detection of abnormalities, empowering healthcare providers to initiate timely interventions. As a result, the application of smart implants is poised to significantly improve treatment outcomes for breast cancer patients, ultimately contributing to better rates of survival and quality of life.

## 3 Limitations

Breast cancer diagnosis has improved over the years, but there are still some gaps that require further investigation. The barrier remained in creating unified diagnostic protocol where multiple imaging (including biosensors, sensor arrays and soon AI) can be merged. Our current continuing challenge is to increase the sensitivity and specificity of mammography, particularly in dense breast tissues as well developing more accurate image quality and diagnostic precision for MRI and US. The sensors currently used for diagnosis have low penetration depth, higher degree of which can give high performance. Besides, the sensors may be integrated to detect over all breast areas for superior results or fabricated and movable in circular axis. They are not very suitable for this diagnosis, like LM35 sensors and mostly they are tested on the breast phantoms. In addition, the development of new biomarkers and multiplex biosensors for multiple detection is required to provide more precise and personalized diagnosis. In AI and deep learning algorithms our challenges would be image variance as well at morphological changes, that needs very efficient in coding the models that can withstand images out of variety of datasets. It’s also critical to investigate wearable technology for continuous monitoring, lessen the need for invasive procedures, and advance non-invasive ways for early detection. Furthermore, it is critical to develop affordable, user-friendly diagnostic techniques and to enhance patient comfort and accessibility, particularly in environments with limited resources. The need for creative and comprehensive methods to enhance breast cancer diagnosis and patient care is highlighted by these research gaps.

## 4 Conclusions and future perspectives

This review has provided a comprehensive analysis of the standard and emerging techniques used for breast cancer (BC) diagnosis. Early detection remains crucial for improving treatment outcomes and survival rates. The study has explored various diagnostic approaches, including advanced imaging techniques such as reflective optical imaging devices (ROIDs), microwave imaging (MWI), automated breast ultrasound (ABUS), and infrared imaging technology (IIT). Additionally, we have highlighted innovative biosensors, including piezoelectric sensors, near-infrared sensors, and digital temperature sensors, each offering unique advantages such as non-invasiveness, enhanced sensitivity, and improved detection accuracy. Despite these advancements, several challenges persist, including the need to enhance diagnostic accuracy, patient comfort, and cost-effectiveness. Many existing technologies, while promising, still require further validation, optimization, and accessibility improvements to ensure widespread clinical adoption. By improving diagnostic precision and reducing false positives, artificial intelligence (AI) has the potential to completely transform the early detection of breast cancer. A subset of deep learning algorithms called Convolutional Neural Networks (CNNs) have shown remarkable performance in image analysis, more accurately detecting tumors in mammograms and ultrasounds than conventional techniques. Large datasets can be processed by AI-driven models to find subtle patterns that humans might miss, resulting in earlier and more accurate diagnoses. Additionally, combining AI with wearable technology—like biosensors and smart bras—allows for continuous data collection and real-time monitoring, which improves patient outcomes and provides individualized risk assessments. Future developments in these technologies will probably concentrate on improving AI algorithms for increased breast cancer screening sensitivity and specificity.

As breast cancer detection methods continue to evolve, wearable and integrated technologies are expected to play a transformative role in early diagnosis and continuous monitoring. One notable innovation in development is the smart bra insert equipped with ultrasound sensors, designed to provide real-time, continuous monitoring of breast tissue. This device detects abnormalities, such as lumps or irregularities, using embedded ultrasound technology, potentially reducing reliance on traditional hospital-based screenings. By integrating these sensors into a comfortable and user-friendly bra design, this technology aims to improve accessibility, encourage proactive health monitoring, and facilitate early detection. Devices like the iTBra have undergone clinical trials to compare performance with standard mammography. Results showed that smart bras could detect anomalies earlier in high-risk populations. Such wearable innovations could significantly reduce late-stage diagnoses and enhance patient outcomes by offering convenient, at-home breast health tracking.

The future of breast cancer detection will likely involve a multi-modal approach, combining AI-driven imaging, advanced biosensors, and wearable technologies to create a more efficient, non-invasive, and personalized diagnostic framework. As research progresses, optimizing these technologies and seamlessly integrating them into routine healthcare practices will be critical in the ongoing fight against breast cancer. Decentralized, continuous breast cancer care with increased precision and privacy may be made possible by emerging technologies like federated AI frameworks, smart fabrics, and microneedle-based biosensors. According to current research, improved mechanical flexibility, multifunctionality, and wireless communication capabilities are anticipated in next-generation biosensing platforms—essential characteristics for a smooth transition into wearable technology and remote healthcare ecosystems (264). Continued interdisciplinary collaboration, clinical trials, and technological refinements will be essential to enhancing early detection methods and ultimately improving patient survival rates worldwide.
